# ACAT2 suppresses the ubiquitination of YAP1 to enhance the proliferation and metastasis ability of gastric cancer via the upregulation of SETD7

**DOI:** 10.1038/s41419-024-06666-x

**Published:** 2024-04-26

**Authors:** Mengmeng Zhang, Fenglin Cai, Jiamei Guo, Siya Liu, Gang Ma, Mingzhi Cai, Rupeng Zhang, Jingyu Deng

**Affiliations:** 1 Department of Gastric Surgery, Tianjin Medical University Cancer Institute & Hospital, National Clinical Research Center for Cancer, Key Laboratory of Cancer Prevention and Therapy, Tianjin, Tianjin Key Laboratory of Digestive Cancer, Tianjin’s Clinical Research Center for Cancer, Tianjin, 300060 PR China; 2 Department of Biochemistry and Molecular Biology, The Province and Ministry Co-sponsored Collaborative Innovation Center for Medical Epigenetics, School of Basic Medical Sciences, Tianjin Medical University, Tianjin, 300060 PR China

**Keywords:** Gastric cancer, HIPPO signalling, Invadopodia, Ubiquitylation

## Abstract

The contributions of aberrantly expressed metabolic enzymes to gastric cancer (GC) initiation and progression have been widely appreciated in recent years. Acetyl-CoA acetyltransferase 2 (ACAT2) is one member of the acetyl- CoA thiolase family. Previous studies demonstrated that ACAT2 either promotes or suppresses tumor progression in different conditions. However, the function and mechanisms of ACAT2 in GC remain unknown. We found that the expression of this enzyme was significantly increased in GC tissues compared with normal counterparts, which prompted us to further investigate the roles of this protein in GC biology. In vitro functional studies showed that ACAT2 knockdown markedly halted the proliferation and the motility of GC cells; these functions favoring malignant phenotypes of GC cells were further validated in animal experiments. Mechanistically, ACAT2 depletion significantly reduced the transcription of SETD7, which is a histone methyltransferase and plays critical roles in GC cells. We found that the pro-tumoral functions of ACAT2 were largely dependent on SETD7. Moreover, SETD7 decreased the ubiquitination level of Yes-associated protein 1 (YAP1), thereby protecting YAP1 from proteasome degradation. Increased YAP1 protein expression remarkably activated the YAP1/TAZ-TEAD1 signaling pathway, which further boosted the malignant phenotypes in GC cells. In conclusion, these findings highlight the pro-tumoral functions and molecular underpinnings of ACAT2 in GC cells, and suggest that ACAT2 could be a promising target in GC treatment.

## Introduction

Gastric cancer is the fifth most commonly diagnosed cancer, the third most common cause of cancer-related deaths worldwide and remains a major global health problem [1]. GC is a malignancy of high aggressiveness with a heterogeneous nature, the aetiology and precise treatment of which remains to be explored [2]. Numerous investigations have clarified the genetic basis of GC while also discovering biomarkers to predict prognosis and response to treatment [3]. A few biomarkers have been employed as treatment targets in advanced GC [4]. However, the therapeutic outcomes remain disappointing. An increasing number of studies on GC have focused on lipid metabolism, a necessity for tumorigenesis and progression, since alterations in intracellular and extracellular metabolites have profound effects on gene expression, cell differentiation and tumour formation [5]. The change in fatty acid metabolism, which is thought to play a major role in cancer progression, is notable [6, 7]. Multiple investigations have shown that abnormal fatty acid metabolism promotes the occurrence, progression and metastasis of GC [8–10].

Acetyl-CoA acetyltransferase (ACAT) consists of a family of two universal metabolic enzymes located in the cytoplasm (ACAT2) and mitochondria (ACAT1) [11, 12]; among these enzymes, ACAT1 has been shown to promote the tumour growth and metastasis of MDA-MB-231 breast cancer cells [13] and is highly expressed in prostate cancer tissue [14]. ACAT2, also known as cytoplasmic acetyl-CoA thiolase, catalyses two molecules of acetyl-CoA to form an acetoacetyl-CoA and a CoA, which is an enzyme that plays a crucial role in lipid metabolism [15, 16], including fatty acid β-oxidation and de novo cholesterol synthesis [17]. ACAT2 deficiency usually leads to inherited organic aciduria which is diagnosed clinically and molecularly [18–20]. ACAT2 promotes radioresistance in oesophageal squamous cell carcinoma (ESCC) and high expression of ACAT2 is significantly correlated with lower overall survival [21]. And Wang et al. found that the expression of ACAT2 is significantly increased in epithelial ovarian cancer cells and high expression of ACAT2 is linked to advanced disease stage, chemotherapy resistance and shorter survival time, suggesting that ACAT2 may be an independent prognostic factor for epithelial ovarian cancer [22]. However, the role of abnormal expression of ACAT2 in GC remains unknown.

The involvement of ACATs in critical cellular function is now becoming clearly demonstrated due to their catalytic function in isoleucine degradation, ketolysis, ketogenesis and fatty acid oxidation [23, 24]. ACATs may contribute to epigenome programming [24] by providing substrates such as acetyl-CoA and other metabolites that can be used by enzymes involved in modulating histone posttranslational modifications (PTMs) [25–27]. These PTMs may further participate in controlling the expression of genes and the conduction of signalling pathways [28, 29], which is commonly occurs in GC.

In our study, we found that ACAT2 was highly expressed in GC tissues and indicated poor prognosis in GC patients. In addition, according to the RNA-seq data, ACAT2 regulates the expression of SETD7 and activates the YAP1/TAZ-TEAD1 axis, which may be a potential target for the treatment of patients with GC.

## Results

### Aberrantly upregulated ACAT2 expression was associated with the poor prognosis of GC

**Fig. 1:**
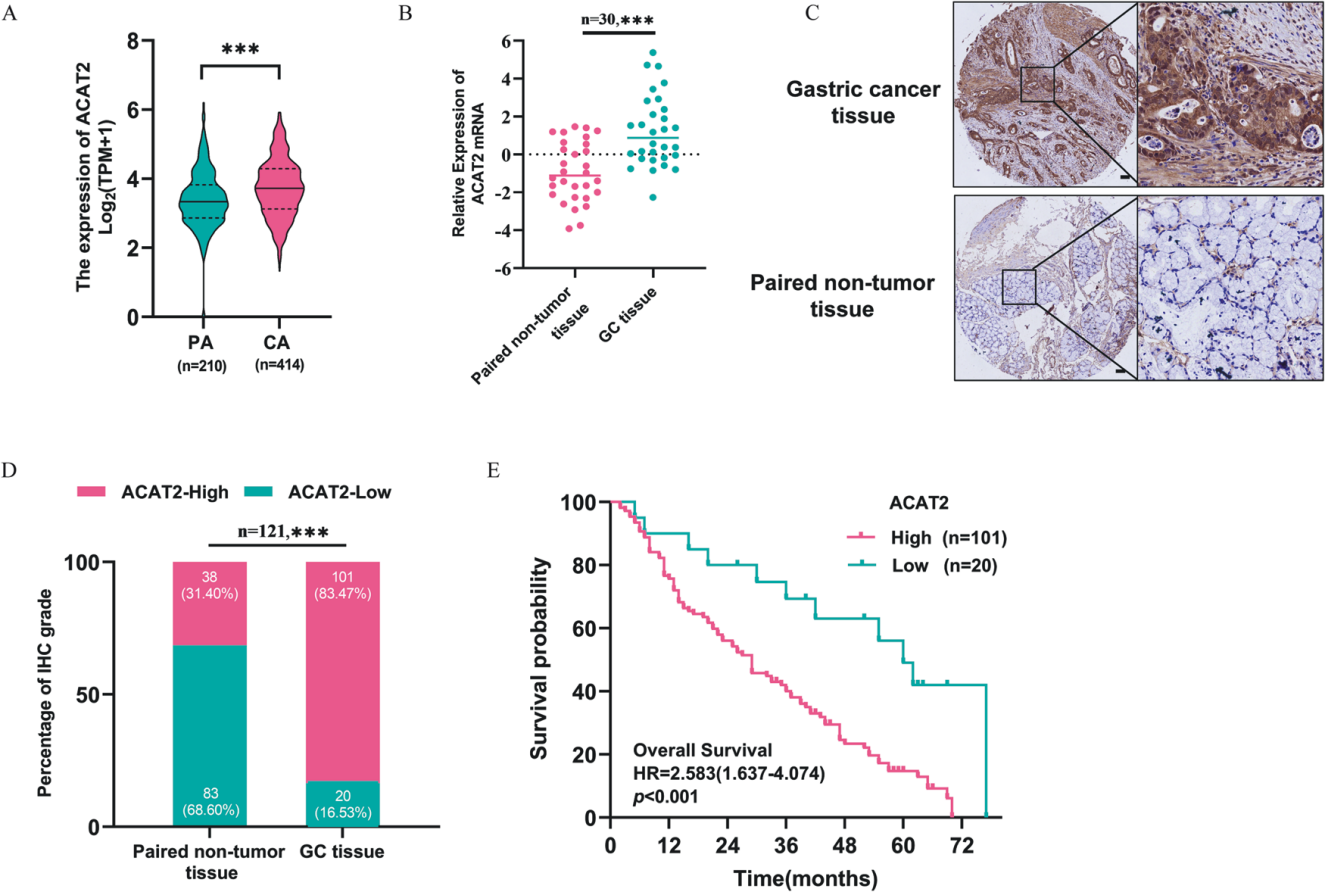
Aberrantly upregulated ACAT2 expression was associated with the poor prognosis of GC. **A** The ACAT2 mRNA level was significantly upregulated in GC tissues (*n* = 414) compared with that in normal tissues (*n* = 210) from the TCGA GC database (*p* < 0.001). **B** ACAT2 mRNA expression is markedly elevated in internal GC tissues (*n* = 30), as measured by qPCR. **C** ACAT2 staining in GC tissues is stronger than that in normal tissues. Representative IHC images are shown here. Scale bar, 100 μm. **D** ACAT2 staining was scored (0–12) and the ACAT2 protein level was markedly increased in GC samples relative to paired nontumor samples (*n* = 121) (*p* < 0.001). **E** Higher ACAT2 expression is indicative of a poorer overall survival rate in institutional patients (*p* < 0.01).

To investigate the role of ACAT2 in GC, the expression level of ACAT2 in GC tissues was examined. ACAT2 mRNA levels were found to be significantly increased in carcinoma tissues (CA) (*n* = 414) when compared with those in paracarcinoma tissue (PA) (*n* = 210) in the GC cohort from The Cancer Genome Atlas (TCGA) database (*p* < 0.001) (Fig. 1A). In the institutional GC tissues, ACAT2 mRNA expression was found to be noticeably higher than that in paired nontumor tissues (*n* = 30, *p* < 0.001) (Fig. 1B). Then, the immunohistochemistry (IHC) assay on 121 pairs of in-house GC and paired adjacent nontumor tissues indicated that the cell cytoplasm and nucleus had positive ACAT2 staining (Fig. 1C). The ACAT2 staining intensity ranged from 0 to 12, and GC samples showed noticeably higher staining than adjacent nontumor samples (*p* < 0.001) (Fig. 1D). Furthermore, patients with higher ACAT2 expression had larger tumour sizes, a greater likelihood of lymph node metastasis and a more advanced pTNM stage (Table 1). In addition, patients with lower ACAT2 expression had much longer overall survival (OS) than those with higher ACAT2 expression (*p* < 0.001) (Fig. 1E). As a result of multivariate regression analysis, ACAT2 was found to be an independent prognostic factor for OS. (*p* = 0.047) (Table 2).

**Table 1:** Analysis of ACAT2 expression in GC tissues and associated clinicopathological factors.

Characteristics	ACAT2		*P* value
	Low	High	
Age, years			0.442
<65	15	64	
≥65	5	37	
Gender			0.179
Male	17	58	
Female	3	33	
Size, cm			0.017*
≤4 cm	10	23	
>4 cm	10	78	
Lymph node metastasis (pN stage)			0.005**
N0	8	12	
N1–N3	12	89	
Tumour location			0.146
Upper third	1	22	
Middle third	2	11	
Lower third	14	44	
More than 2/3 stomach	3	24	
pTNM classification			0.001**
I	2	0	
II	7	17	
III	11	84	
Depth of invasion (pT stage)			0.271
T2–T3	4	16	
T4	11	90	
Lauren type			1.000
Intestinal	5	25	
Diffuse	15	76	

*pTNM classification* is defined using the American Joint Committee on Cancer (AJCC) Staging System, 8th Edition. Asterisks stand for *P* values as follows: *P < 0.05; **P < 0.01.

**Table 2:** Univariate and multivariate Cox regression analyses for overall survival of gastric cancer patients.

Predictor	Univariate Analysis		Multivariate Analysis	
	HR (95% CI)	*P*	HR (95% CI)	*P*
Age, years				
≥65 vs <65	1.546 (1.014–2.359)	0.043*		
Gender				
Female vs Male	1.458 (0.953–2.230)	0.082		
Size, cm				
>4 cm vs ≤4 cm	2.055 (1.267–3.335)	0.004*	1.770 (1.089–2.876)	0.021*
Tumour location				
Middle 1/3 vs Upper 1/3	0.966 (0.463–2.016)	0.926		
Lower 1/3 vs Upper 1/3	0.781 (0.449–1.360)	0.382		
More than 2/3 stomach vs Upper 1/3	0.984 (0.525–1.843)	0.959		
pTNM classification				
II vs I	1.584 (0.206–12.201)	0.659		
III vs I	3.791 (0.522–27.541)	0.188		
Depth of invasion				
T4 vs T2–T3	0.999 (0.532–1.879)	0.999		
Lymph node metastasis (pN stage)				
N1–N3 vs N0	2.596 (1.376–4.895)	0.003**	2.083 (1.093–3.971)	0.026*
Lauren type				
Diffuse vs Intestinal	1.371 (0.837–2.247)	0.211		
Expression of ACAT2				
High vs Low	2.932 (1.508–5.701)	0.002**	2.003 (1.010–3.970)	0.047*

*pTNM classification* is defined using the American Joint Committee on Cancer (AJCC) Staging System, 8th Edition. Asterisks stand for *P* values as follows: *P < 0.05; **P < 0.01.

### ACAT2 expression potentially enhances the proliferative ability of GC cells

**Fig. 2:**
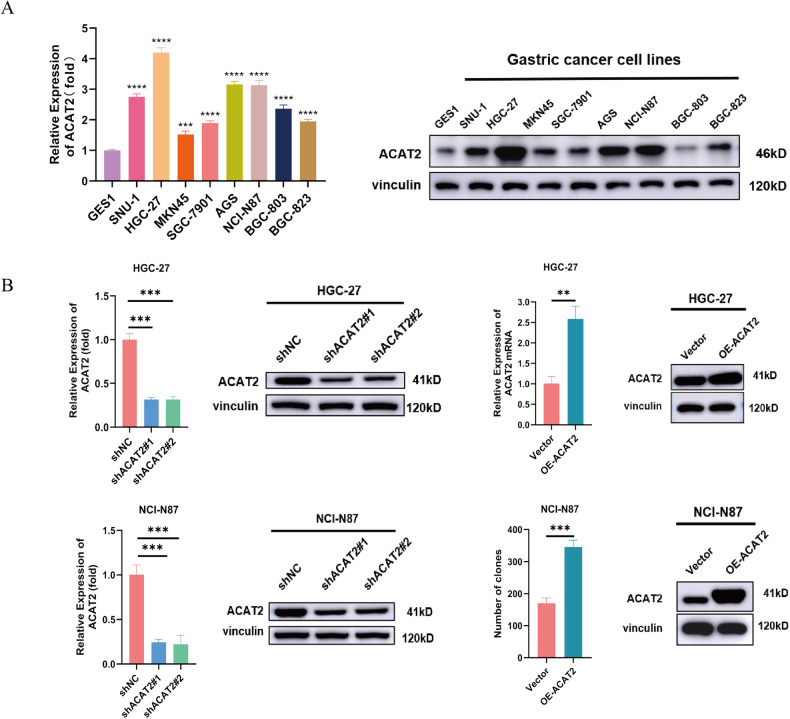

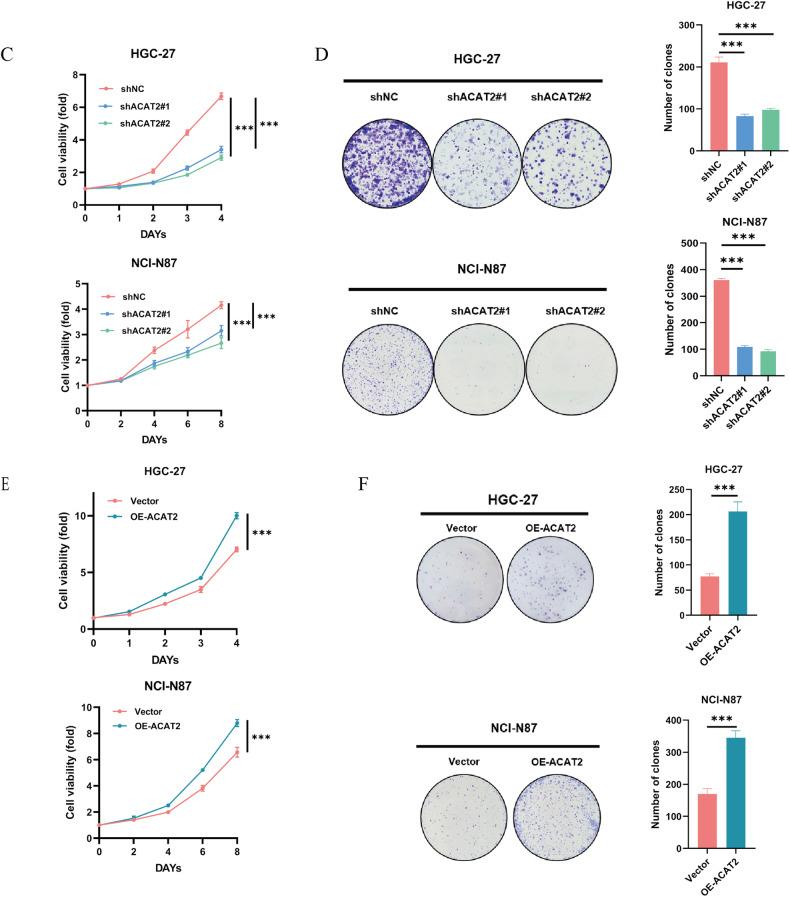

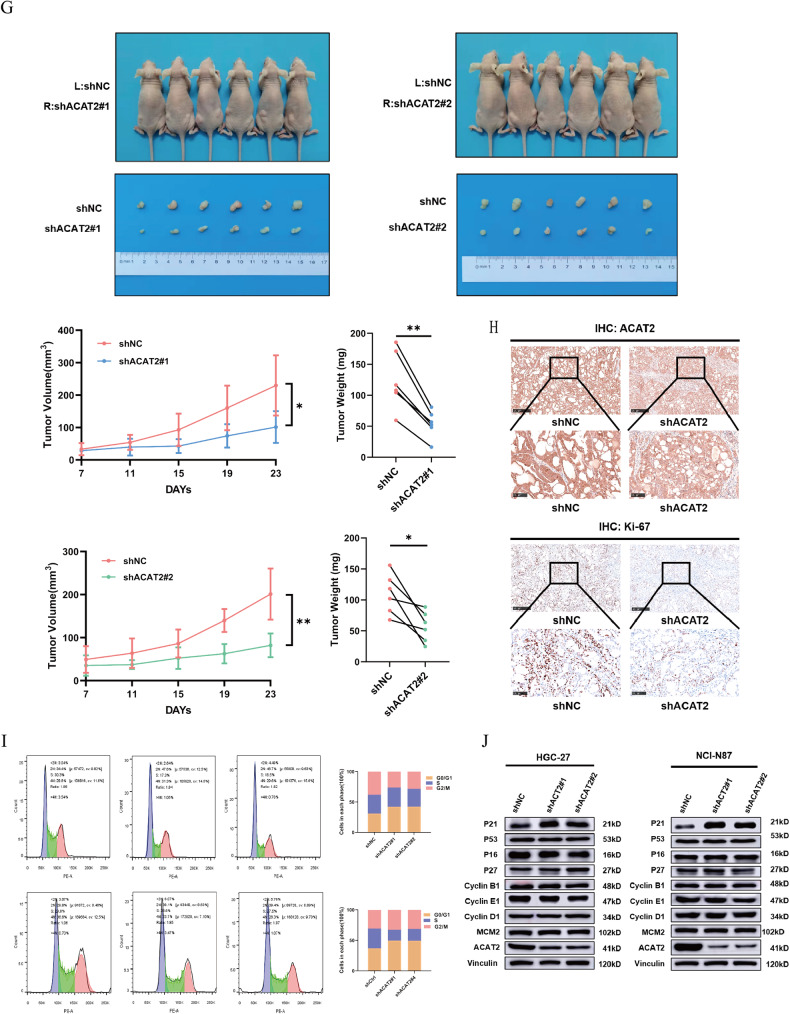
ACAT2 expression potentially enhances the proliferative ability of GC cells. **A** ACAT2 expression levels were examined by qPCR assay and western blotting assay in the immortalised human gastric epithelial cell line GES-1 and a set of GC cell lines. **B** The knockdown (left panel) and re-expression (right panel) efficiency of ACAT2 in HGC-27 and NCI-N87 cells was verified by qPCR assay and western blotting assay. **C** ACAT2 depletion markedly inhibits proliferation in HGC-27 and NCI-N87 cells, as verified by the CCK-8 assay; **D** and colony formation assay. **E** Cellular growth of HGC-27 and NCI-N87 cells from the vector and ACAT2-restored groups was assayed using the CCK8 assay; **F** and colony formation assay. **G** ACAT2 knockdown inhibits the growth of tumours in vivo, and tumour growth curves as well as tumour weight show the suppressive effect of ACAT2. **H** IHC shows that Ki-67 staining was markedly weaker in tumour masses originating from ACAT2-depleted NCI-N87 cells. **I** The cell cycle was blocked in G1/S phase in response to knocking down ACAT2, as determined by flow cytometry analysis of HGC-27 and NCI-N87 cells. Data are expressed as the means ± SEMs for at least three independent experiments. **J** The expression of many proteins closely related to the cell cycle was examined by western blotting.

This study evaluated the role of ACAT2 in the malignant biological behaviour of GC. As proven above, increased ACAT2 expression was associated with large tumour size and advanced pN stage. Before conducting the functional research, we discovered that the levels of the ACAT2 protein were markedly higher in eight GC cell lines than in GES-1 cells (Fig. 2A). Given that ACAT2 was overexpressed in GC, we next sought to address the effect of ACAT2 on cell propagation by using shRNAs to deplete endogenous ACAT2 expression in HGC-27 and NCI-N87 cells and simultaneously transducing ACAT2-expressing (OE-ACAT2) or control lentivirus (Vector) into HGC-27 and NCI-N87 cells, respectively (Fig. 2B). CCK-8 and colony formation assays showed that ACAT2 depletion significantly inhibited HGC-27 and NCI-N87 cell growth and reduced the number of colonies in comparison to those of control cells (Fig. 2C, D). In contrast, ACAT2 overexpression significantly promoted HGC-27 and NCI-N87 cell growth (Fig. 2E), as well as that in MKN45 and BGC-823 cells (Fig. S1A–C) and increased the number of colonies in comparison to those of control cells in HGC-27 and NCI-N87 cell (Fig. 2F). Then, the control and ACAT2 knockdown NCI-N87 cells were injected subcutaneously into immunocompromised mice to test the growth of GC cells in vivo. As a result of reduced ACAT2 expression in NCI-N87 cells, the growth rate was significantly decreased, as was the weight of the harvested tumour mass. (Fig. 2G). Moreover, IHC confirmed that ACAT2-depleted NCI-N87 tumour masses manifested significantly weaker Ki-67 staining than the tumour masses derived from the control cells (Fig. 2H). Collectively, these findings demonstrate that ACAT2 contributes to the proliferation of GC cells in vitro and tumour growth in vivo. Furthermore, to explore the mechanism by which ACAT2 promotes the proliferation of GC cells, we detected the cell cycle through flow cytometry and found that both the HGC-27 and NCI-N87 cell cycles were blocked in G1/S phase after ACAT2 knockdown. (Fig. 2I). Additionally, we measured the expression of many proteins closely related to the cell cycle and found that the expression level of p21 (also known as cyclin-dependent kinase inhibitor 1A, CDKN1A) was significantly increased after ACAT2 knockdown, which corresponded to the detection results of the cell cycle (Fig. 2J). We believe that ACAT2 knockdown can block the cell cycle in G1/S phase and inhibit the malignant proliferation of GC cells.

### ACAT2 expression potentially enhances the motility of GC cells

**Fig. 3:**
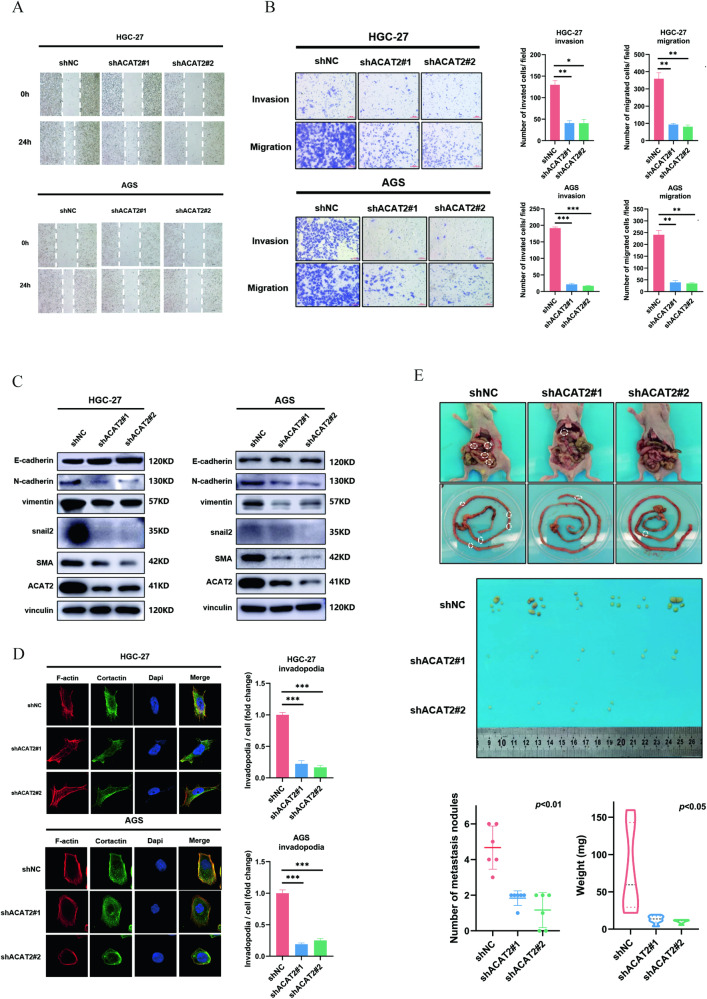
ACAT2 expression potentially enhances the motility of GC cells. **A**, **B** ACAT2 depletion attenuated the invasion and migration ability of HGC-27 and AGS cells, as measured by wound healing and transwell assays (scale bar, 100 μm). **C** ACAT2 knockdown inhibited the EMT process in HGC-27 and AGS cells. **D** Invadopodia formation assay (magnification 630×) with immunofluorescence staining for F-actin (red), cortactin (green) and 4′,6-diamidino-2-phenylindole (DAPI) (blue). **E** Representative images of intraperitoneal implant metastasis in mice, body and tumour weights and tumour numbers (*n* = 6, *p* < 0.05), indicating that ACAT2 depletion attenuates GC cell migration in vivo.

To elucidate the functions of ACAT2 in promoting the motility of GC cells, the effects of ACAT2 on the migration and invasion of HGC-27 and AGS cells were detected by wound healing and transwell assays (Fig. 3A, B). Then, we examined the expression of related indicators of the epithelial-mesenchymal transformation (EMT) process (E-cadherin, N-cadherin, vimentin, SMA and snail2), which are closely related to tumour metastasis. The results showed that the knockdown of ACAT2 could upregulate E-cadherin and downregulate N-cadherin, vimentin, SMA and snail2, indicating that ACAT2 could promote the EMT process of GC cells (Fig. 3C). Furthermore, an invadopodia formation assay was employed and showed that ACAT2 depletion significantly reduced the formation of invadopodia (Fig. 3D) in HGC-27 and AGS cells. Collectively, these results indicated that ACAT2 enhanced the motility of GC cells. After that, the control and the ACAT2 knockdown HGC-27 cells were injected intraperitoneally into immunocompromised mice to test the metastases of GC cells in vivo. As a result of reduced ACAT2 expression in HGC-27 cells, the number of metastases was significantly decreased, as was the weight of the harvested tumour mass. (Fig. 3E).

### SETD7 might be considered a potential key downstream molecule of ACAT2

**Fig. 4:**
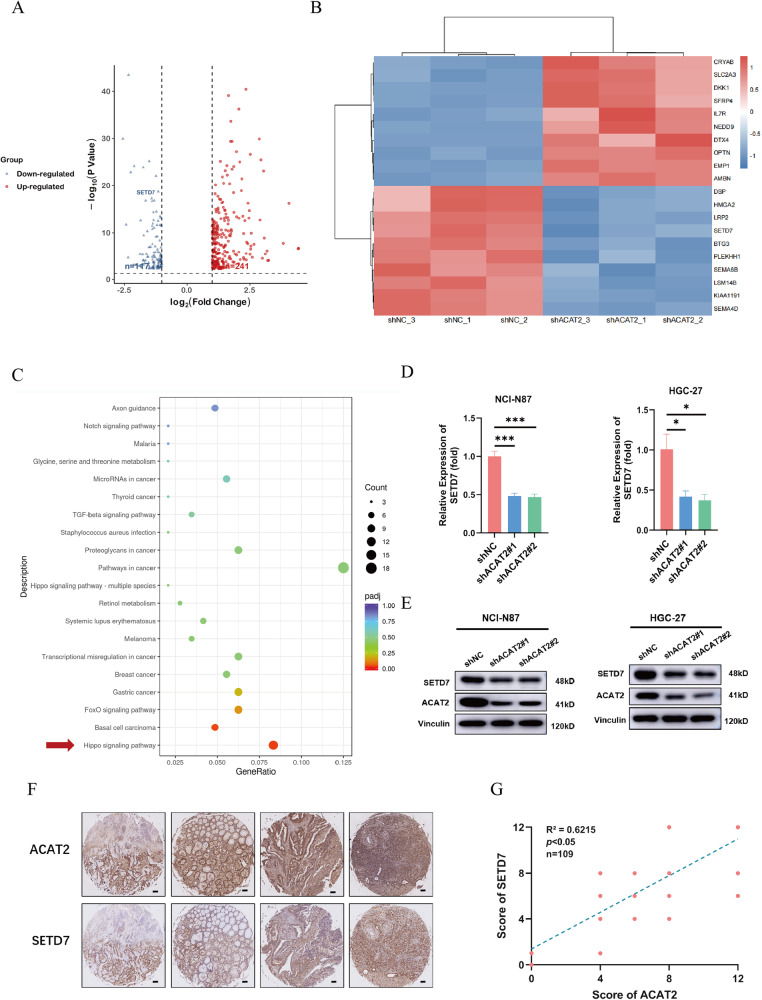
SETD7 might be considered a potential key downstream molecule of ACAT2. **A** The differentially expressed genes (DEGs) from RNA-seq data (GSE246567) are depicted in the volcanic maps, with 358 genes altered. (|log2(FC)| > 1 and *p* < 0.05). **B** The cluster of heatmap identified the top 10 up- and downregulated genes in HGC-27 according to *p* value. **C** The Hippo pathway was regulated in ACAT2 knockdown HGC-27 cells according to KEGG analysis of RNA-seq data (GSE246567). **D** SETD7 expression levels were significantly downregulated after ACAT2 knockdown in HGC-27 and NCI-N87 cells by qPCR assay; **E** and western blotting assay. **F** Representative images of ACAT2 and SETD7 stained in the same instinct GC tissues. Scale bar, 100 μm. **G** and the staining scores were positively correlated (*n* = 109) (*R*^2^ = 0.6215, *p* < 0.05).

To further explore the downstream molecular regulatory mechanism of ACAT2, we investigated the gene expression profiles of ACAT2-deficient (shACAT2) and control (shNC) HGC-27 cells. Knockdown of ACAT2 led to 358 differentially expressed genes (DEGs) in HGC-27 cells, which is displayed in the volcano maps (Fig. 4A). The top 10 up- and downregulated genes are displayed in a heatmap (Fig. 4B). Then, we further conducted Kyoto Encyclopedia of Genes and Genomes (KEGG) pathway enrichment analysis based on the RNA-seq data (GSE246567) and found that multiple tumour-related pathways were activated, among which the Hippo signalling pathway was the most affected one by ACAT2 deficiency (Fig. 4C). Considering that, among the DEGs, SETD7 is involved in the regulation of different pathways involved in KEGG enrichment, including the Hippo signalling pathway [30], FoxO signalling pathway [31, 32] and TGF-beta signalling pathway [33], we believe that SETD7 plays a hub role in the regulation of downstream molecular mechanisms. According to earlier studies, the SETD7 gene promotes the occurrence and propagation of cancers and may play different roles in tumour development, for example promoting the metastasis of triple-negative breast cancer by Yin Yang 1 lysine methylation [34] and promoting bladder cancer progression and immune escape via the STAT3/PD-L1 cascade [35]. Nevertheless, what part of SETD7 acts in the carcinogenesis of GC remains unknown. Then, we continued our investigation into the relevant gene SETD7. qPCR and immunoblot assays confirmed that knockdown of endogenous ACAT2 expression elicited significantly decreased SETD7 expression in HGC-27 and NCI-N87 cells (Fig. 4D, E). We discovered a positive correlation between the staining intensity of SETD7 and ACAT2 in the GC tissues (*n* = 109, *R*^2^ = 0.6215, *p* < 0.05) via immunohistochemical staining (Fig. 4F, G). These results collectively demonstrated that ACAT2 directly increased the expression of SETD7 in GC cells.

### SETD7 knockdown inhibits the proliferation and migration of GC cells both in vitro and in vivo

**Fig. 5:**
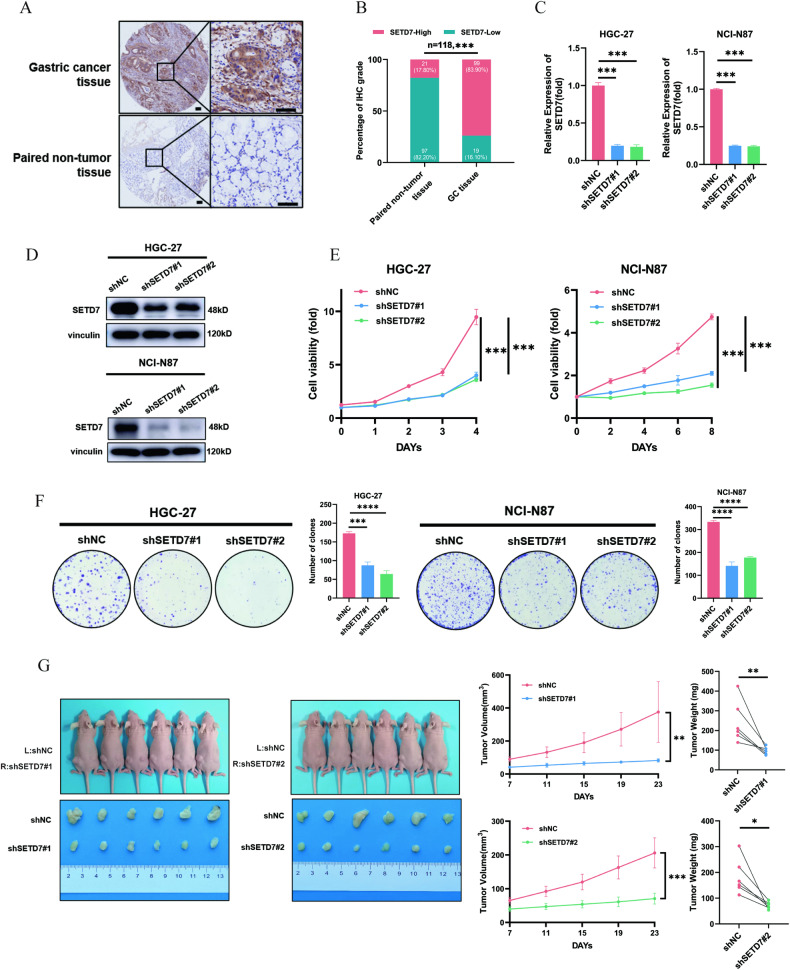

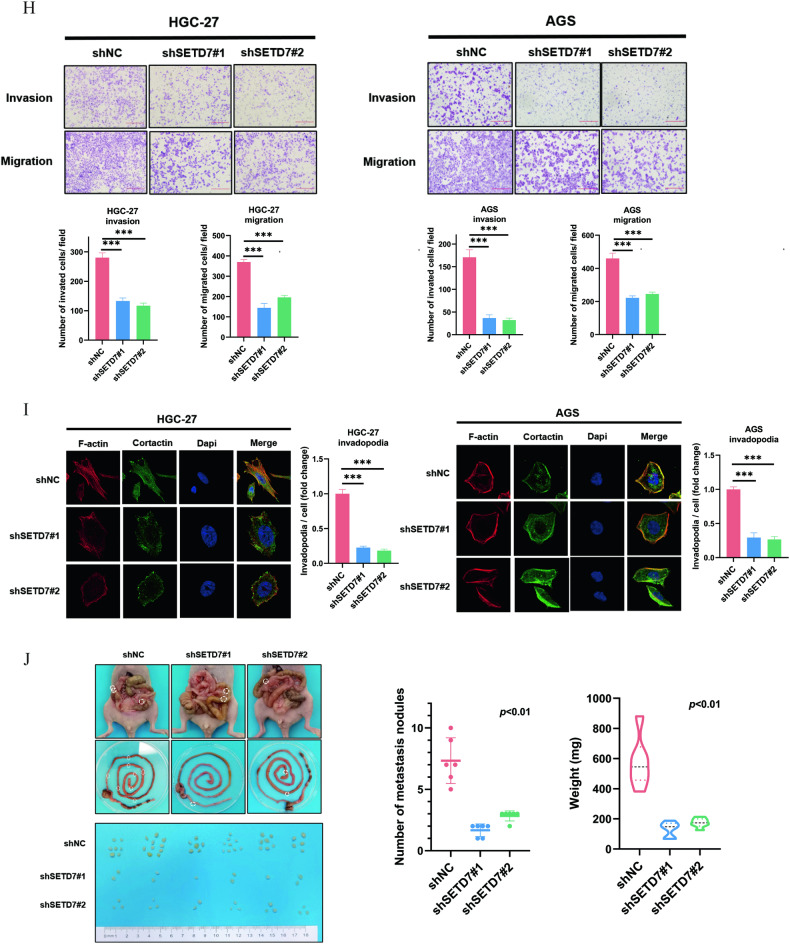
SETD7 knockdown inhibits the proliferation and migration of GC cells in vitro and in vivo. **A** SETD7 staining in GC tissues is stronger than that in matched nontumor tissues. Representative IHC images are shown here. Scale bar, 100 μm. **B** SETD7 staining was scored (0–12), and the SETD7 protein level was markedly increased in GC samples relative to paired nontumor samples (*n* = 118) (*p* < 0.001). **C** The endogenous expression of SETD7 in HGC-27 and NCI-N87 cells was decreased using shRNAs verified by qPCR assay, (**D**) and western blotting assay. **E** SETD7 downregulation markedly inhibits proliferation in HGC-27 and NCI-N87 cells, as verified by CCK8 assay; **F** and colony formation assay. **G** SETD7 knockdown inhibits the growth of tumours in vivo, and tumour growth curves as well as tumour weight show the inhibitory effect of SETD7. **H** SETD7 depletion markedly inhibits motility in HGC-27 and AGS cells, as detected by transwell assay (magnification 100×); **I** and invadopodia formation assay (magnification 630×). **J** Representative images of intraperitoneal implant metastasis in mice and the weights and numbers of tumours (*n* = 6, *p* < 0.05), indicating that SETD7 knockdown inhibits the migration of GC cells in vivo.

To explore the role of SETD7 in GC, SETD7 protein expression was measured via IHC staining in in-house GC tissues and nontumor tissues, showing that the expression level of SETD7 in GC tissues is significantly higher than that in paired nontumor tissues (Fig. 5A, B). Then, we used shRNAs to knock down the endogenous expression of SETD7 in HGC-27 and NCI-N87 cells. qPCR and immunoblotting verified the knockdown efficiency of SETD7 (Fig. 5C, D). Proliferation function investigations demonstrated that HGC-27 and NCI-N87 cells with diminished endogenous SETD7 expression delayed growth and impaired colony formation in vitro when compared with those in control cells (Fig. 5E, F). Moreover, SETD7 deletion decreased the growth rate of NCI-N87 cells in vivo (Fig. 5G), and IHC confirmed that SETD7-depleted NCI-N87 tumour masses manifested significantly weaker Ki-67 staining than the tumour masses derived from the control cells (Fig. S2A, B). In addition, transwell assays and invadopodia formation assays showed damage to the motility of HGC-27 and AGS cells (Fig. 5H, I). In addition, the diminished weight and number of metastatic tumours compared with those of the control group of intraperitoneal implant metastasis experiments proved that downregulated SETD7 inhibited the metastasis of HGC-27 cells in vivo (Fig. 5J).

### The malignant effects of ACAT2 rely on the high expression of SETD7 in GC cells

**Fig. 6:**
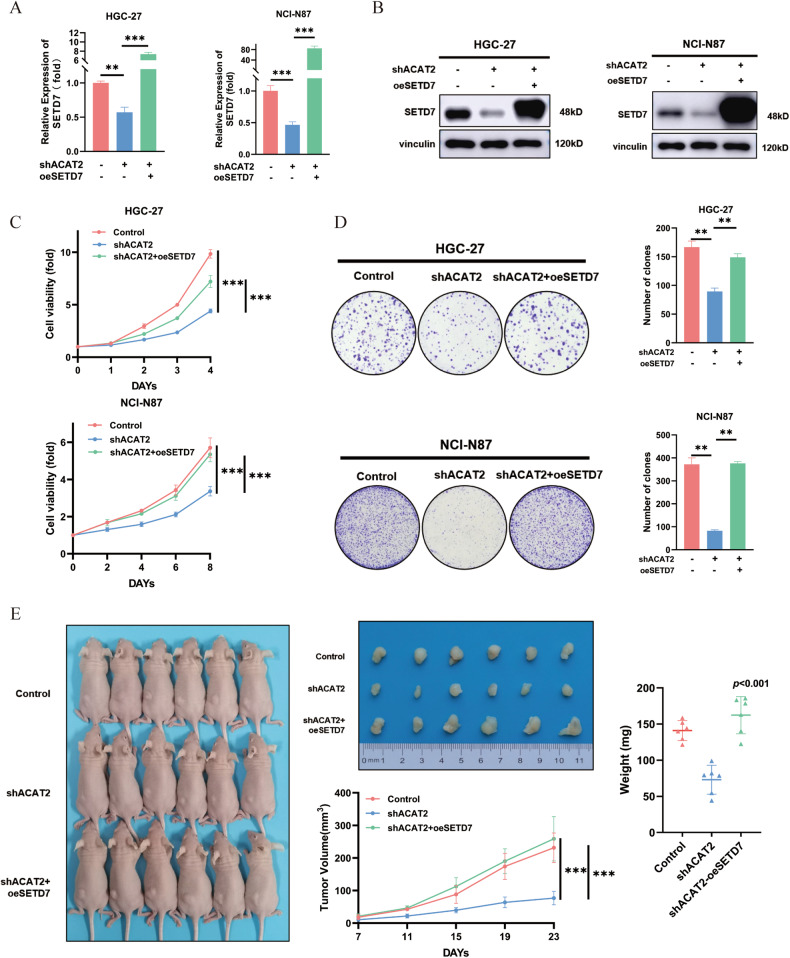

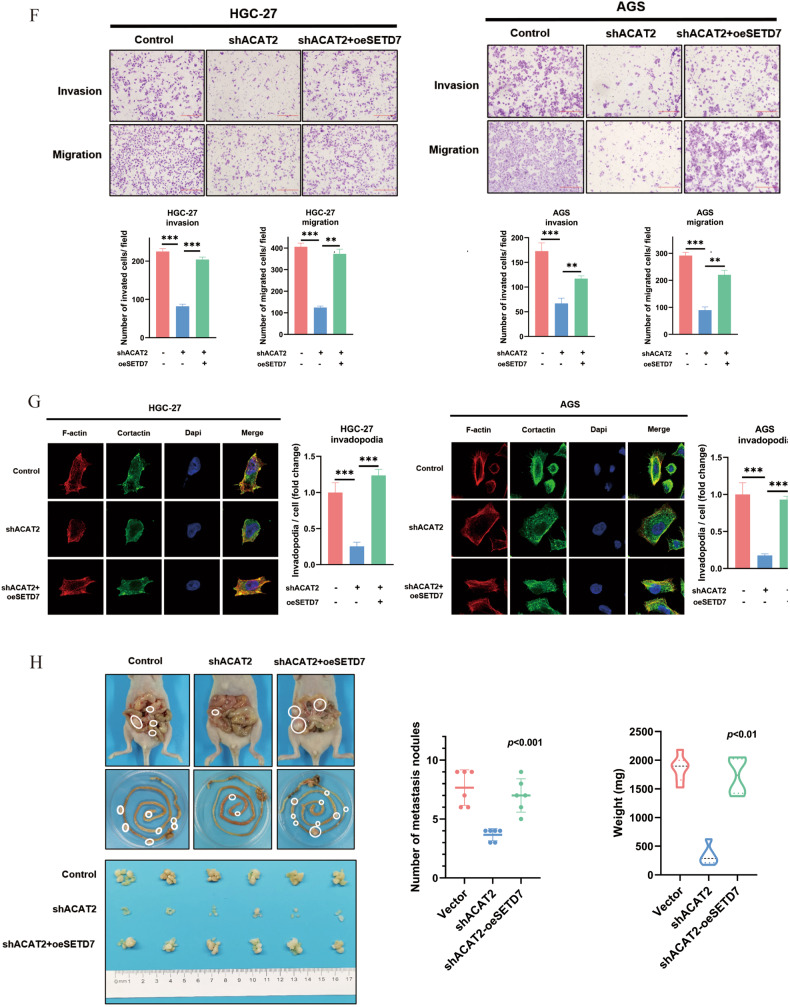
The malignant effects of ACAT2 rely on the high expression of SETD7 in GC cells. **A** SETD7 expression was elevated in ACAT2-deficient HGC-27 and NCI-N87 cells, as verified by qPCR assay; **B** western blotting assay. **C** Elevated SETD7 expression reignited the proliferation of ACAT2-deficient HGC-27 and NCI-N87 cells in vitro, as verified by CCK8 assay; **D** and colony formation assay. **E** Subcutaneous tumour formation experiment in mice, of which tumour growth curves and tumour weight show that overexpressed SETD7 expression reignited GC cell proliferation in vivo. **F** SETD7 overexpression significantly restored the motility of ACAT2-deficient HGC-27 and AGS cells, as detected by transwell assay (magnification 100×); **G** and invadopodia formation assay (magnification 630×). **H** Upregulated SETD7 expression reversed intraperitoneal implant metastasis in ACAT2-deficient HGC-27 cells.

To determine whether SETD7 expression was vital for the proliferation and metastasis-promoting activity of ACAT2, we overexpressed exogenous SETD7 in the presence of control shRNA or ACAT2 knockdown (Fig. 6A, B). Increased SETD7, according to functional analysis, reversed the inhibition of proliferation triggered by ACAT2 knockdown in HGC-27 and NCI-N87 cells in vitro and in vivo (Fig. 6C–E), as well as the inhibition of metastasis caused by ACAT2 knockdown in HGC-27 and AGS cells in vitro and in vivo (Fig. 6F–H). Overall, these findings showed that SETD7 expression was needed, at least in part, for the malignant effects of ACAT2 in GC cells.

### SETD7 stabilises YAP1 to strengthen the YAP/TAZ-TEAD1 axis

**Fig. 7:**
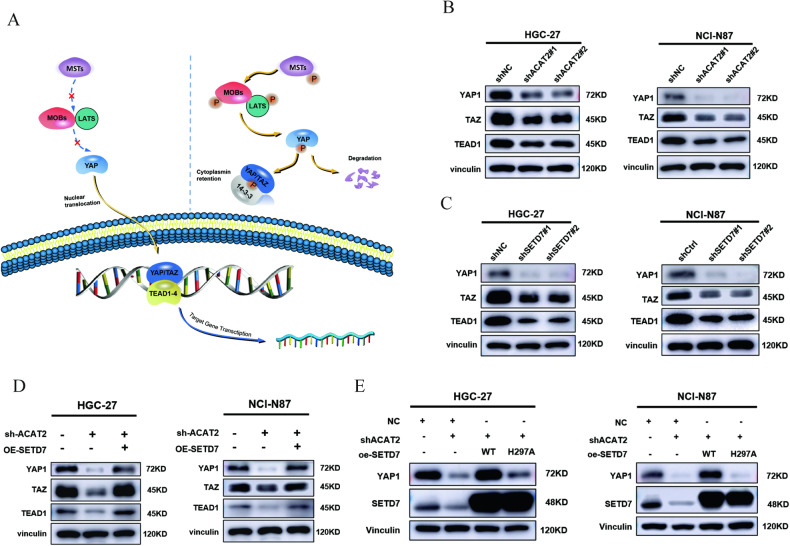

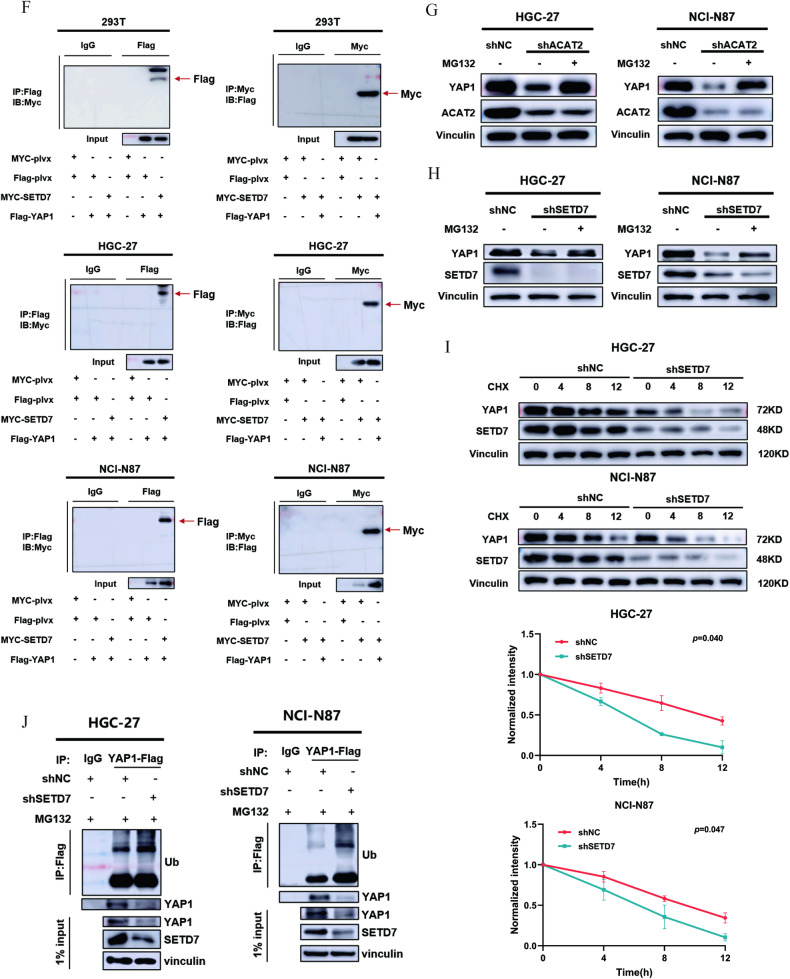
SETD7 stabilises YAP1 to strengthen the YAP/TAZ-TEAD1 axis. **A** Schematic diagram of the Hippo signalling pathway. **B** western blot analysis of YAP1, TAZ and TEAD1 in HGC-27 and NCI-N87 cells with ACAT2 knockdown; **C** and SETD7 knockdown. **D** The expression of YAP1, TAZ, and TEAD1 was restored when SETD7 was overexpressed in HGC-27 and NCI-N87 cells with ACAT2 knockdown. **E** The immunoblotting assay showed that high expression of wild-type SETD7 in ACAT2-deficient HGC-27 and NCI-N87 cells reversed the change in YAP1 protein levels, while the upregulation of the SETD7 mutant (SETD7H297A) failed to achieve a similar reversal effect. **F** The direct binding of SETD7-Myc and YAP1-Flag fusion proteins was confirmed by Co-IP experiments in HEK-293T, HGC-27 and NCI-N87 cells. **G** The immunoblotting assay showed that MG132 mitigated the inhibitory effect of ACAT2 on YAP1; **H** and the inhibitory effect of SETD7 on YAP1 in HGC-27 and NCI-N87 cells. **I** HGC-27 and NCI-N87 cells expressing SETD7 shRNA were exposed to 100 μm CHX at the indicated time points for 0, 4, 8 and 12 h. YAP1 protein expression was analysed by immunoblotting and quantified by ImageJ software. **J** The poly-ubiquitination level of endogenous YAP1 in GC cells stably transfected with shNC or shSETD7 was assessed by in vivo ubiquitination assay. 1% input of cell lysates was used to assess the expression of YAP1 and SETD7.

As mentioned above, the Hippo signalling pathway was the most activated signalling pathway by ACAT2 deficiency based on the RNA-seq data (GSE246567). Yes-associated protein (YAP), transcriptional coactivator with PDZ-binding motif (TAZ, also known as WWTR1) and TEA domain family members (TEAD1-4) constitute the main effect axis of the Hippo signalling pathway (Fig. 7A), which promotes the development of GC. In our study, immunoblot assays confirmed that the protein levels of the YAP1/TAZ-TEAD1 axis were markedly decreased in GC cells with ACAT2 or SETD7 deficiency (Fig. 7B, C). Overexpression of exogenous SETD7 in the presence of control shRNA or ACAT2 knockdown restored the protein levels in HGC-27 and NCI-N87 cells (Fig. 7D); however, the mutation SETD7^H297A^ without methyltransferase activity failed to restore the YAP1 level in GC cells (Fig. 7E). We believe that the regulatory effect of ACAT2 on the YAP/TAZ-TEAD1 axis depends on the methyltransferase activity of SETD7. As shown in Fig. 7F, the interaction between endogenous SETD7 and YAP1 in HEK-293T and GC cells was verified by a coimmunoprecipitation assay. Furthermore, the inhibition of YAP1 by ACAT2 and SETD7 was nearly abrogated by the proteasome inhibitor MG132 (Fig. 7G, H). Additionally, the results of the CHX pulse-chase assay showed that SETD7 knockdown shortened the half-life of YAP1 and accelerated its degradation (Fig. 7I). Then, we validated that the ubiquitination level of YAP1 was greatly elevated in GC cells stably transfected with shSETD7 (Fig. 7J), indicating that SETD7 prevented the degradation of YAP1 through the ubiquitin-proteasome system (UPS).

## Discussion

Lipid metabolism reprogramming is now widely recognised as a crucial step in the development of GC, which initiates and propagates damage that leads to cancer cell proliferation and metastasis [36, 37]. In recent years, acetyl-coenzyme A acetyltransferase (ACAT), also known as acetoacetyl-CoA thiolase, has emerged, of which the two primary members ACAT1 and ACAT2 play an important role in fatty acid metabolism [15]. Currently, research on the ACAT family in oncology mainly focuses on ACAT1. Little is known about the role of ACAT2 in tumours. ACAT2 is found within the endoplasmic reticulum (ER) of various tissues, including the liver, intestine and adipose tissue [38]. Studies have shown that ACAT2 may suppress the progression of clear cell renal cell carcinoma (ccRCC) by promoting apoptosis of tumour cells and inhibiting epithelial-mesenchymal transition (EMT) [39, 40]. Intriguingly, we presented both clinical and experimental evidence that ACAT2 was upregulated in GC in our study. Clinicopathological data analysis of GC patients from our hospital indicated that the high expression of ACAT2 was closely associated with later pN stage, larger tumour size and lower survival rate. Further analysis revealed that elevated ACAT2 expression in patients with GC was an independent risk factor. Similarly, the same conclusion was confirmed in cell function and animal experiments. Obviously, ACAT2 plays a completely opposite role in GC and ccRCC. The discrepancy might result from the various ways that different cancer types employ their metabolic machinery, which is brought on by genetic background differences.

Metabolic rewiring and epigenetic remodelling are closely linked processes, and they mutually regulate each other in cancers [41]. In our data, the expression of histone–lysine N-methyltransferase (SETD7) is closely related to ACAT2, and the rescue experiment proved that SETD7 was regulated by ACAT2. SETD7, a histone methylation modifier, that methylates histone and nonhistone substrates [42, 43], plays a crucial role in PTMs. It contributes significantly to digestive system cancer [44] by affecting the methylation or demethylation of its histone or nonhistone substrates to induce gene silencing or activation, and further regulates carcinogenesis, proliferation [45], EMT [46], invasion and migration of cancers [47]. One study showed that SETD7 expression was significantly increased in GC tissues compared to that in matched nontumor gastric tissues, indicating that SETD7 may contribute to GC aggressiveness and metastasis [46], contrary to another study that suggested that SETD7 inhibits the growth and invasion of GC cells [48]. It is worth noting that research on SETD7 in GC is still ongoing, and more studies are needed to fully elucidate its precise role and mechanism of action. The findings thus far suggest that SETD7 may be involved in GC progression, but further research is necessary to validate these observations and determine the therapeutic potential of targeting SETD7 in GC treatment.

SETD7 regulates a variety of cancer-related processes, in a tissue-type and signalling context-dependent manner [49]. SETD7 methylates crucial molecules in numerous significant signalling pathways, including STAT3 [50], p53 [51], HIF-1α [52] and PARP1 [53]. In addition, research has demonstrated that SETD7 inhibits YAP transport to the nucleus and reduces the transcription of YAP target genes via YAP monomethylation at K494 [30]. In our study, we found that the expression levels of YAP1 protein were significantly decreased after SETD7 knockdown in GC cells, which was reversed by rescue experiments, while SETD7 mutants (SETD7^H297A^) did not decrease the expression levels of YAP1 protein, indicating that SETD7 inhibits ubiquitination and degradation of YAP1 depending on its methyltransferase activity. Interestingly, there was no significant alteration in the nuclear localisation of YAP1 by SETD7 through nuclear-cytoplasmic isolation of the protein in GC (data not shown), which was opposed to earlier research [30]. As such, we investigated additional potential regulatory mechanisms of SETD7 on YAP1 in GC. Our further investigation demonstrated that SETD7 promotes the ubiquitination and degradation of YAP1 in GC cell lines. Due to the great diversity of cancer cells, our perspective is meant to be provocative, not universal. Nevertheless, our data may reveal a novel regulatory mechanism of SETD7 on YAP1.

There are some limitations in this study. First, to a large extent, ACAT2 affects the production and utilisation of acetyl-CoA, which serves as the hub for central carbon metabolism, linking metabolism, catabolism and energy production in eukaryotic cells [54]; therefore, the transcriptional regulation of acetyl-CoA by SETD7 requires further investigation. Second, SETD7 was first identified as a histone H3-lysine 4-specific (H3K4) methyltransferase [42], of which the SET domain is needed for catalysis, with histidine 297 being the critical site for its methyltransferase activity [55]. We have proposed a new possible mechanism by which SETD7 regulates YAP1 in GC. SETD7 methylates YAP1, thereby inhibiting the ubiquitination and degradation of YAP1 in GC cell lines. As a crucial biomarker of cancers, which is now a hot spot in current research in cancer, the methylation site of YAP1 by SETD7 deserves further investigation. We will explore this problem in our future research.

In conclusion, our findings provide evidence that ACAT2 plays a significant role in promoting the proliferation and metastasis of GC cells in vivo and in vitro, and the molecular mechanisms underlying this phenotype are that the downstream gene SETD7 of ACAT2 methylates YAP1 and suppresses its ubiquitination and degradation, activating the YAP1/TAZ-TEAD1 axis that contributes to GC cell malignancy. Our results characterise the carcinogenic function of ACAT2 in GC cells and provide insight into the possible intricate roles of ACAT2 in GC pathogenesis and therapy, which need to be fully evaluated.

## Materials and methods

### Patients and tissue samples

The tissue microarrays (TMAs, Cat No. T14-501TMA1-3) used in this study were composed of 121 pairs of tumour and paired adjacent non-tumour tissues, which were retrieved from Tianjin Medical University Cancer Hospital (Tianjin, China), Xijing Hospital of Air Force Medical University (Xi’an, China) and Renji Hospital of Shanghai Jiao Tong University School of Medicine (Shanghai, China) between August 2004 and December 2007, These TMAs were engineered by Shanghai Outdo Biotech Company (Shanghai, China). Follow-up was performed every 3–6 months and completed in September 2012. The median was 30.0 months (range: 2–77 months).

Additionally, GC and paired adjacent non-tumour gastric mucosa specimens (*n* = 30) were collected from patients receiving curative gastrectomy in 2021 at Tianjin Medical University Cancer Hospital (Tianjin, China) to detect ACAT2 mRNA level. All experiments used these samples were approved by the Institutional Research Ethics Committee of Tianjin Medical University Cancer Institute and Hospital (Tianjin, China).

### Cell lines and cell culture

HGC-27, MGC-803, BGC-823, SGC-7901 and human immortalised gastric epithelial cells (GES-1) were from the National Infrastructure of Cell Line Resource (Beijing, China). Human GC cell lines (NCI-N87, SNU-1, and AGS) were purchased from American Type Culture Collection (VA, USA). MKN45 cells was a gift from Prof. Hui Li from Department of Gastrointestinal Cancer Biology at Tianjin Medical University Cancer Institute and Hospital, Tianjin, China. HEK293T cells were offered by Prof. Zhihua Liu from the National Cancer Center/Cancer Hospital, Beijing, China. All cell lines were cultured in RPMI-1640 supplemented with 10% fetal bovine serum (FBS, Newzerum, Christchurch, New Zealand), except for HEK293T, which were cultured in DMEM containing 10% FBS and AGS, cultured in F12K with 10% FBS. Cells were maintained in a cell incubator with 5% CO_2_ at 37 °C. All cells with no more than 20 continuous passages were used in this study. All cell lines were verified as Mycoplasma negative.

### Antibodies and reagents

Rabbit anti-ACAT2 (1:1000) (ab131215) and rabbit anti-P21 (1:1000) (ab109520) were purchased from Abcam. Rabbit anti-SETD7 antibody(1:1000) (24840-1-AP), rabbit anti-P16-INK4A(1:1000) (10883-1-AP), rabbit anti-P27 (1:1000)(25614-1-AP), rabbit anti-Cyclin B1 (1:1000)(55004-1-AP), rabbit anti-Cyclin E1(1:1000)(11554-1-AP), mouse anti-Cyclin D1(1:1000)(60186-1-Ig), rabbit anti-MCM2(1:1000)(10513-1-AP), rabbit anti-Cortactin (1:400)(11381-1-AP) and rabbit anti-SMA (1:1000)(14395-1-AP), mouse anti-Vimentin(1:4000)(60330-1-Ig) were purchased from Proteintech. Rabbit anti-Snail2 (1:1000) (121235) was purchased from Brickell Biotech, Inc. Antibodies against YAP1(1:1000) (A1002), TAZ (1:1000) (A23034), TEAD1(1:1000) (A5218) were purchased from AB clonal. Rabbit anti-vinculin (1:1000) (E1E9V), rabbit anti-flag antibody (1:1000) (2272S), rabbit anti-myc antibody (1:1000) (14793S), rabbit anti-p53 (7F5) (1:1000)(2527S), rabbit anti-E-Cadherin (24E10) (1:1000)(3195S), rabbit anti-N-Cadherin (D4R1H) XP®(1:1000)(13116S) and mouse anti-ubiquitin (1:1000) (#3936) were purchased from Cell Signalling Technology.

MG132 (HY-13259), Cycloheximide (CHX) (HY-12320) and Cell Counting Kit-8 (CCK-8, HY-K0301) were purchased from MedChemExpress (Shanghai, NJ, USA).

### Plasmids, lentivirus production and generation of stable cell lines

The pLVX-IRES-G418 vector and pSIH-H1-puro vector were generously provided by Prof. Zhihua Liu from the National Cancer Center/Cancer Hospital, Beijing, China. Lentivirus was produced by simultaneously introducing the indicated lentiviral vectors, psPAX2 (Plasmid #12260) and pMD2.G (Plasmid #12259) into HEK293T cells. Short hairpin RNA (shRNA) for stable transfection was designed and synthesised to downregulate ACAT2 and SETD7. Empty pLVX-IRES-neo vector and pSIH1-H1-puro vector were used as negative controls (shNC and vector). The shRNA sequences were as follows: 5′- CCAGCCAATGCTTCAGGAATA-3′ (shACAT2#1), 5′- CCAAGCTAAAGCCTTACTTTC-3′ (shACAT2#2), 5′- TAAGTGTAAACTCCCTGGC-3′ (shSETD7#1) and 5′-AGGAAGGCTCTTCTAGCAATA -3′(shSETD7#2). The full-length cDNA samples of human ACAT2-Flag (NM_005891.3), SETD7-Myc (SETD7^WT^-Myc) and YAP1-Flag (NM_006106.5) were subcloned and inserted into a PLVX-IRES-G418 vector. To abrogate the enzymatic activity of SETD7, we replaced the histidine with alanine at SETD7 (SETD7^H297A^). Cells were incubated with the indicated lentivirus and polybrene (1 μl/ml) for 24 h. G418 (400 μg/ml) or puromycin (2 μg/ml) was used to establish stable cell populations. The transfection experiment was carried out using Lipofectamine ^TM^ 2000 (Thermo Fisher, CN2514240).

### Gastric cancer dataset analysis

The analysis of ACAT2 and SETD7 mRNA expression in TCGA GC cohort was performed using online integrated tools (https://www.xiantao.love/).

### RNA extraction and RT-qPCR

Total RNA was extracted from cultured cells or GC/normal tissue samples using RNAiso plus (Takara Bio, Shiga, Japan). The cDNAs were generated using GoScript™ Reverse Transcription Kit (Promega, Madison, WI, USA). all genes were determined on the QuantStudio 5 real-time PCR system (Applied Biosystems, Foster City, CA, USA) using TB Green Premix Ex TaqTM II (TaKaRa). β-actin was used for data normalisation. The −ΔCt method was used to determine ACAT2 expression in tissues, and the 2^−ΔΔCt^ method was used to evaluate the expression of the indicated genes in GC cell lines. The qPCR primer sequences in this study were listed in Supplementary Table 1.

### RNA-sequencing and analysis

Total RNA was extracted from ACAT2-knockdown and control GC cells. The sequence and the data analysis were conducted by LC-Bio (Hangzhou, Zhejiang, China). Differentially expressed genes (DEGs) were defined as fold change >1 and *p* < 0.05, and then Gene ontology (GO) enrichment and KEGG pathway enrichment analyses were done. All services were provided by LC Biotech Corporation (Hangzhou, China). The data are deposited under GSE246567 in GEO database.

### CCK-8 assay and colony formation assay

CCK-8 assays were performed to examine cell proliferation in vitro. Briefly, a total of 1000 HGC-27 cells or 3000 NCI-N87 cells were seeded into 96-well plates in sextuple. CCK-8 (Zeta Life, China) was added to the cell suspension at a ratio of 1:10 and incubated at 37 °C for 2 h. The absorbance at 450 nm was measured by a microplate reader (BioTek) at the indicated time points. For colony formation assay, HGC-27(1 × 10^3^ cells/well) and NCI-N87 cells (1 × 10^4^ cells/well) from the control and test group were seeded in 6-well plates respectively and cultured for around 10 days. The colonies were fixed with methanol for 10 min, stained with 0.1% crystal violet, and counted under microscope.

### Cell cycle assay

To examine the effect of ACAT2 on cell cycle in HGC-27 and NCI-N87 cells, all cells were fixed by 70% alcohol in PBS at 4 °C overnight. After washing with PBS, cells were then incubated with 50 µl mL^−1^ PI solution and 0.5% RNase solution (DOJINDO, C543) in assay buffer in dark at 37 °C for 30 min. After being extensively washed with PBS, cells were resuspended in PBS and analysed by FACS Calibur flow cytometry (BD FACSCanto II, USA).

### Transwell assay and invadopodia formation assay

For the transwell assay, HGC-27 (4 × 10^4^ cells) and AGS (6 × 10^4^ cells) cells were seeded into a transwell chamber precoated with or without Matrigel. Then, 600 µl of medium containing 20% foetal bovine serum was added to the bottom chambers. The transwell cells were incubated at 37 °C for 24 h. Then, the cells were immobilised with methanol for 10 min, stained with 0.1% crystal violet and counted under a microscope.

For the invadopodia formation assay, GC cells (HGC-27, 4 × 10^4^ and AGS, 4 × 10^4^ cells) were plated on 18-mm cover glasses until they adhered to the surface. The cells were fixed and permeabilized with 0.1% Triton X-100. Then, they were blocked with 2% bovine serum albumin (BSA) and incubated with primary antibodies against CTTN at 4 °C overnight and then TRITC Phalloidin (Solarbio, CA1610-300T) at 37 °C for 6 h. The proteins were visualised by incubation with anti-rabbit IgG conjugated to Alexa Fluor 488 (Cell Signaling Technology) for 30 min at room temperature, and the nuclei were stained with DAPI Fluoromount-G® (SouthernBiotech, 0100-20) for another 10 min. All images were captured using an Axio Imager Z2 microscope (Zeiss, Oberkochen, Germany).

To measure the percentage of merged area in each field, identical signal thresholds for FITC fluorescence were set for all images in an experiment and the merged area with FITC signals above the set threshold was measured by ImageJ. The resulting percentage of fusion area was further normalised to the total cell number (counted by DAPI staining for nuclei) in each field. The final mergence index is the average percentage mergence per cell obtained from all three fields. Each experiment was repeated at least three times.

### Protein extraction and western blotting

Cells were washed with pre-chilled PBS buffer and total proteins were extracted with RIPA buffer (Boster Biological Technology, China) supplemented with protease and phosphatase inhibitors (Roche). The protein concentrations were determined using PierceTM BCA protein assay kit (Thermo Fisher Scientific). Denatured proteins were electrophoresed by vertical SDS-PAGE system (Bio-Rad, USA) and transferred to polyvinylidene fluoride (PVDF) membranes. Membranes were blocked with 5% nonfat milk buffer for 1 h and then incubated with primary antibodies overnight at 4 °C. Following washing with TBST, the membrane was incubated with secondary antibody for 1 h and was visualized using chemiluminescence reagent (Thermo Scientific, USA).

### Immunohistochemistry

The tissue microarrays were sent to Shanghai Outdo Biotech Company (Shanghai, China) to evaluate the expression of ACAT2. The antibody concentrations of ACAT2 and SETD7 were 1:800 and 1:600, respectively. The staining intensity of ACAT2(Abcam, ab131215) and SETD7(Proteintech, 24840-1-AP) was estimated by three independent pathologists without knowledge of the clinical data. The staining index (SI) was evaluated by the intensity and proportion of positively stained tumour cells as follows. Scores of staining intensities were: 0, negative; 1, weak; 2, moderate; 3, strong. Scores of positively stained cell proportion were: 0, no positive; 1, <10%; 2, 10–35%; 3, 35–75%; 4, >75%. Using this method, SI with possible scores of 0, 1, 2, 3, 4, 6, 8, 9 and 12 were obtained among the GC samples. An optimal cutoff value was then defined by the median SI score. After that, high and low expression was defined with the optimal cutoff value of 8 for ACAT2 and 6 for SETD7.

### Co-immunoprecipitation (Co-IP) assay

To analyse the binding relationship between SETD7 and YAP1 protein, Co-IP assay was conducted with HEK-293T, HGC-27 and NCI-N87 cells. Total proteins were extracted 72 h after cells were co-transfected with the indicated plasmids, and lysed with 1%NP-40. The protein lysates were incubated with anti- FLAG® M2-Magnetic Beads (Sigma, M8823) or Anti-Myc-tag mAb-Magnetic Beads (Medical & Biological Laboratories, M047-11) or Mouse IgG1 (isotype control)-Magnetic Beads (Medical & Biological Laboratories, M075-11) at 4 °C overnight, collected with magnetic stand, and then washed with 0.05%NP-40 thrice. Finally, the precipitates were collected for subsequent Western blot analysis.

### Animal experiments

All experimental protocols were in accordance with the requirements of the Cancer Institute of Tianjin Medical University and the Animal Care and Use Committee. Female 4-week-old BALB/c nude mice were purchased from Vital River Laboratories (Beijing, China) and housed under specific-pathogen-free (SPF) conditions.

For the subcutaneous injection model, mice were double-blindly and randomly divided into two groups (six per group). NCI-N87 cells (1 × 10^6^ cells) of the control or treated group were suspended in 100 μl of PBS and injected into the dorsal flanks of the mice. The tumour volume was measured every 2 days using callipers. The tumour volume was calculated using the following formula: V = (width^2^ × length) × 0.5. The nude mice were then sacrificed, and their transplanted tumours were removed for other experiments. If the nude mice showed signs of pain during the process of tumour growth, such as significant weight loss, lethargy, or tumour rupture, the nude mice were sacrificed by cervical dislocation. The tumour was collected and weighed at 23 days after implantation. After being photographed, tumours were embedded in paraffin. Paraffin-embedded xenografts were then sliced into serial 6.0 μm sections for haematoxylin and eosin (H&E) staining and IHC staining using anti-Ki67 antibody (Cell Signaling Technology, 9129S, 1:400), anti-ACAT2 (Abcam, ab131215,1:800), or anti-SETD7 antibody (Proteintech,24840-1-AP, 1:600).

For the intraperitoneal implant transfer model, mice were double-blindly and randomly divided into two groups (six per group). HGC-27 cells (5 × 10^6^ cells) in the control or treated group were suspended in 100 μl of PBS and injected into the intraperitoneally into the mice. The weights of the mice were measured every 3 days using electronic scales. The nude mice were then sacrificed, and their transplanted tumours were removed for other experiments. If the nude mice showed signs of pain during the process of tumour growth, such as significant weight loss, lethargy, or pain, the nude mice were sacrificed by cervical dislocation. The tumours were collected and weighed 56 days after the injection. After being photographed, tumours were embedded in paraffin.

### Protein stability and degradation experiment

ACAT2 or SETD7 depletion GC cells and negative control were incubated with CHX (HY12320; Med Chem Express) for 0 h,4 h,8 h,12 h and 24 h at a concentration of 100 μg/mL, and the total protein was extracted for the western blot analysis. SETD7 depletion GC cells (shSETD7) and negative control (shNC) were incubated with 10 μM MG132 (HY-13259; Med Chem Express), and the same amount of dimethyl sulfoxide (DMSO) was added to the control group. Then, 24 h after this treatment, the total protein was extracted for Western blot analysis.

### Ubiquitination assay

To detect endogenous ubiquitination of YAP1, SETD7 depletion GC cells (shSETD7) and negative control (shNC) were incubated with MG132 (10 μM) for 8 h and then lysed by RIPA buffer. Proteins in the cell lysate were immunoprecipitated to isolate ubiquitinated YAP1 with anti-FLAG® M2-Magnetic Beads and the endogenous ubiquitin chains on YAP1 were detected through immunoblotting assay with an antibody against ubiquitin.

### Statistical analysis

Except animal, IHC and HTS assays, all experiments were performed at least twice independently, and all values are expressed as mean ± standard deviation. GraphPad Prism version 8 (San Diego, CA, USA) was used, and tests were performed using Student’s *t* test or *χ*^2^-test unless otherwise specified. *p* < 0.05 was considered statistically significant. Significance levels *p < 0.05; **p < 0.01; ***p < 0.001.

### Supplementary information

Figure suplementary 1

Figure suplementary 2

Figure suplementary 3

Table suplementary 1

Supplementary Figure and Table Legends

Original data file for WesternBlot

Original data file for q-PCR

checklist

## Data Availability

The GC datasets (GSE246567) from the GEO repository database (https://www.ncbi.nlm.nih.gov/gds) was used in this study. The data that support the findings of this study are available from the corresponding author upon reasonable request. All data generated or analysed during this study are included in this published article and its supplementary information files.
