# Supplementary figures and images for: ACAT2 suppresses the ubiquitination of YAP1 to enhance the proliferation and metastasis ability of gastric cancer via the upregulation of SETD7
Source: Cell Death Dis. 2024 Apr 26;15(4):297. doi: 10.1038/s41419-024-06666-x (PMC11053133; doi:10.1038/s41419-024-06666-x)

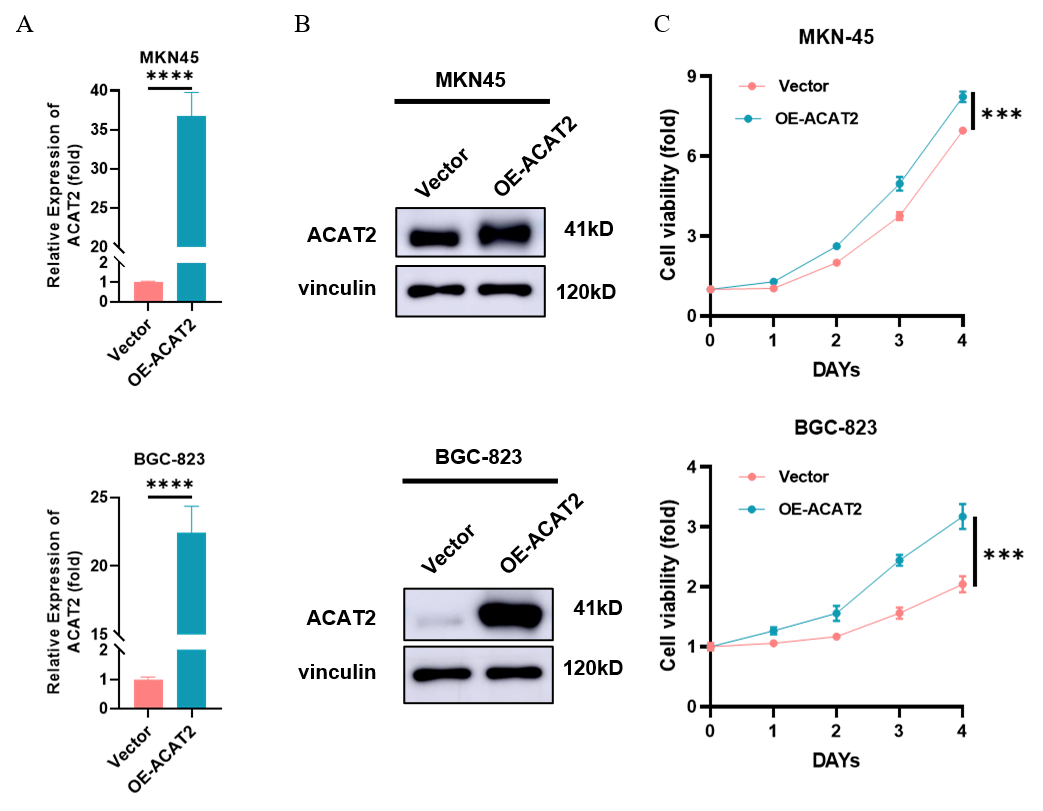

Supplement: Supplementary file 1 — Figure suplementary 1 [file 41419_2024_6666_MOESM1_ESM.tif]

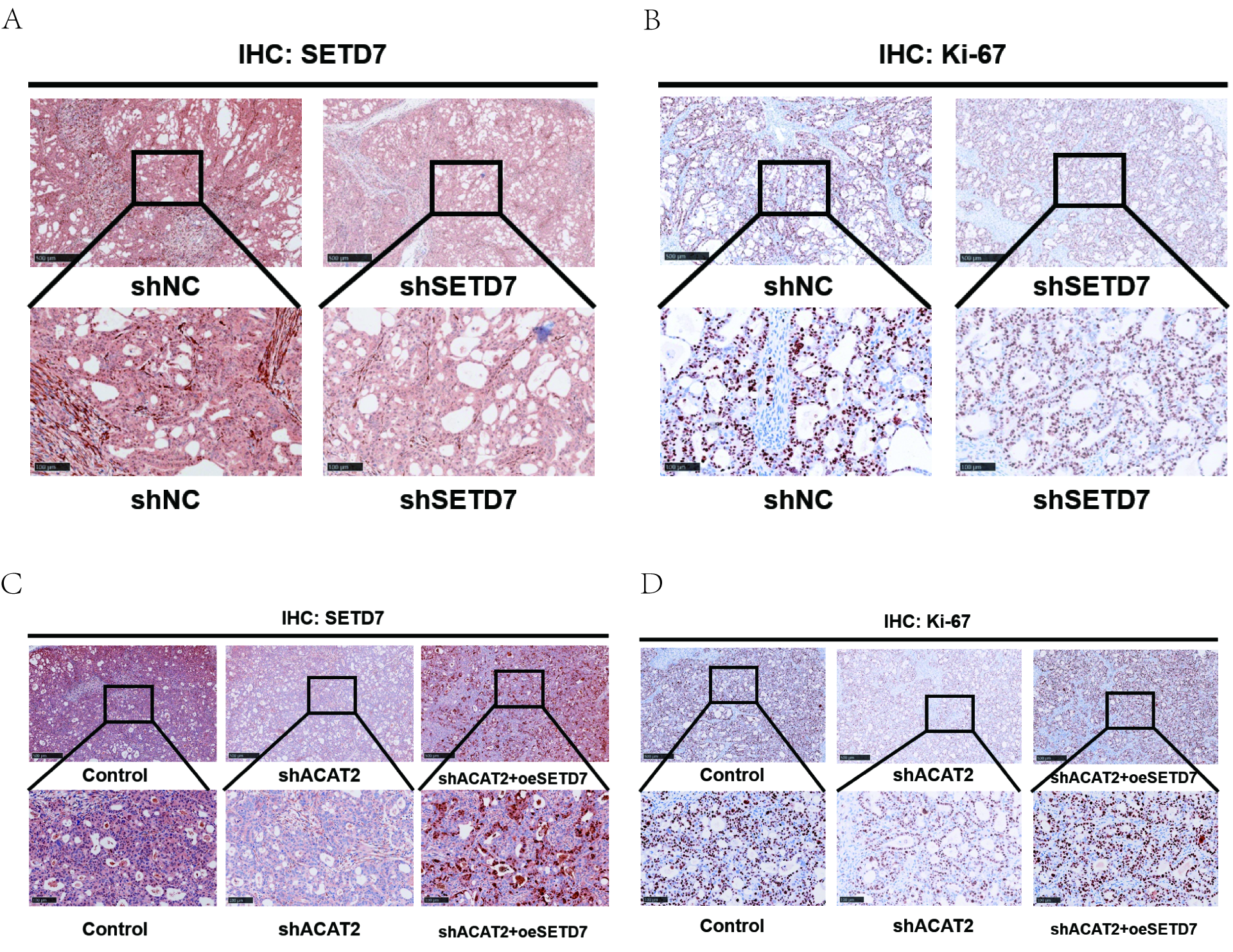

Supplement: Supplementary file 2 — Figure suplementary 2 [file 41419_2024_6666_MOESM2_ESM.tif]

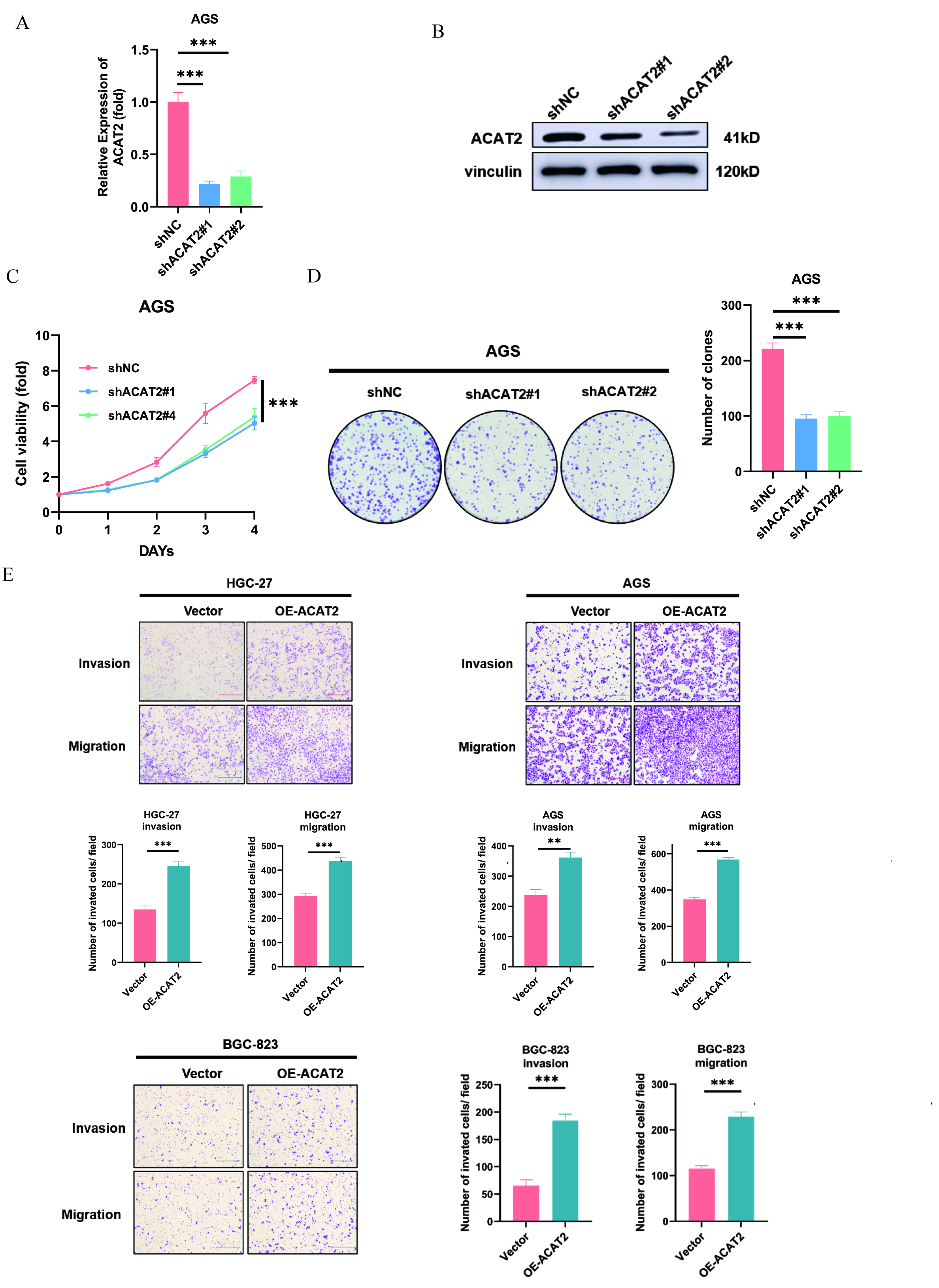

Supplement: Supplementary file 3 — Figure suplementary 3 [file 41419_2024_6666_MOESM3_ESM.tif]
