# Supplementary material for: ACAT2 suppresses the ubiquitination of YAP1 to enhance the proliferation and metastasis ability of gastric cancer via the upregulation of SETD7
Source: Cell Death Dis. 2024 Apr 26;15(4):297. doi: 10.1038/s41419-024-06666-x (PMC11053133; doi:10.1038/s41419-024-06666-x)
Supplement: Supplementary file 4 — Table suplementary 1 [file 41419_2024_6666_MOESM4_ESM.docx]

Supplementary Table 1 qPCR primers used in this study

| **Primer Name** | **Forward Primer Sequence (5' to 3')** | **Reverse Primer Sequence (5' to 3')** |
| --- | --- | --- |
| **ACAT2** | TGGTGCCTTAGCTGCTGTTCCT | GGCTTGTCTAACAGGATTCTGCC |
| **SETD7** | CGTATGTAGACGGAGAGCTGAAC | CTCCTACAAGGCTTCCTCCATC |
| **β-actin** | ATAGCACAGCCTGGATAGCAACGTAC | CACCTTCTACAATGAGCTGCGTGTG |
