# Supplementary material for: ACAT2 suppresses the ubiquitination of YAP1 to enhance the proliferation and metastasis ability of gastric cancer via the upregulation of SETD7
Source: Cell Death Dis. 2024 Apr 26;15(4):297. doi: 10.1038/s41419-024-06666-x (PMC11053133; doi:10.1038/s41419-024-06666-x)
Supplement: Supplementary file 5 — Supplementary Figure and Table Legends [file 41419_2024_6666_MOESM5_ESM.docx]

**Figures S1**

A, B The overexpression efficiency of ACAT2 in MKN45 and BGC-823 cells was verified by qPCR assay and western blotting assay. C ACAT2 overexpression markedly promote proliferation in MKN45 and BGC-823 cells, as verified by the CCK-8 assay.

**Figures S2**

A, B IHC shows that Ki-67 staining was markedly weaker in tumour masses originating from SETD7-depleted NCI-N87 cells. C, D Ki-67 staining of ACAT2 knockdown mouse tumour masses was rescued by SETD7 overexpression.

**Figures S3**

A, B The inherent expression of ACAT2 in AGS cells was diminished using shRNAs, as verified by qPCR and western blotting assays. C, D ACAT2 depletion markedly inhibits proliferation in AGS cells, as verified by CCK-8 and colony formation assays. E ACAT2 overexpression promotes the migration and invasion ability of HGC-27, AGS and BGC-823 cells.

**Table S1** qPCR primers used in this study
