# Supplementary material for: ACAT2 suppresses the ubiquitination of YAP1 to enhance the proliferation and metastasis ability of gastric cancer via the upregulation of SETD7
Source: Cell Death Dis. 2024 Apr 26;15(4):297. doi: 10.1038/s41419-024-06666-x (PMC11053133; doi:10.1038/s41419-024-06666-x)
Supplement: Supplementary file 6 — Original data file for WesternBlot [file 41419_2024_6666_MOESM6_ESM.pdf]

Fig. 2

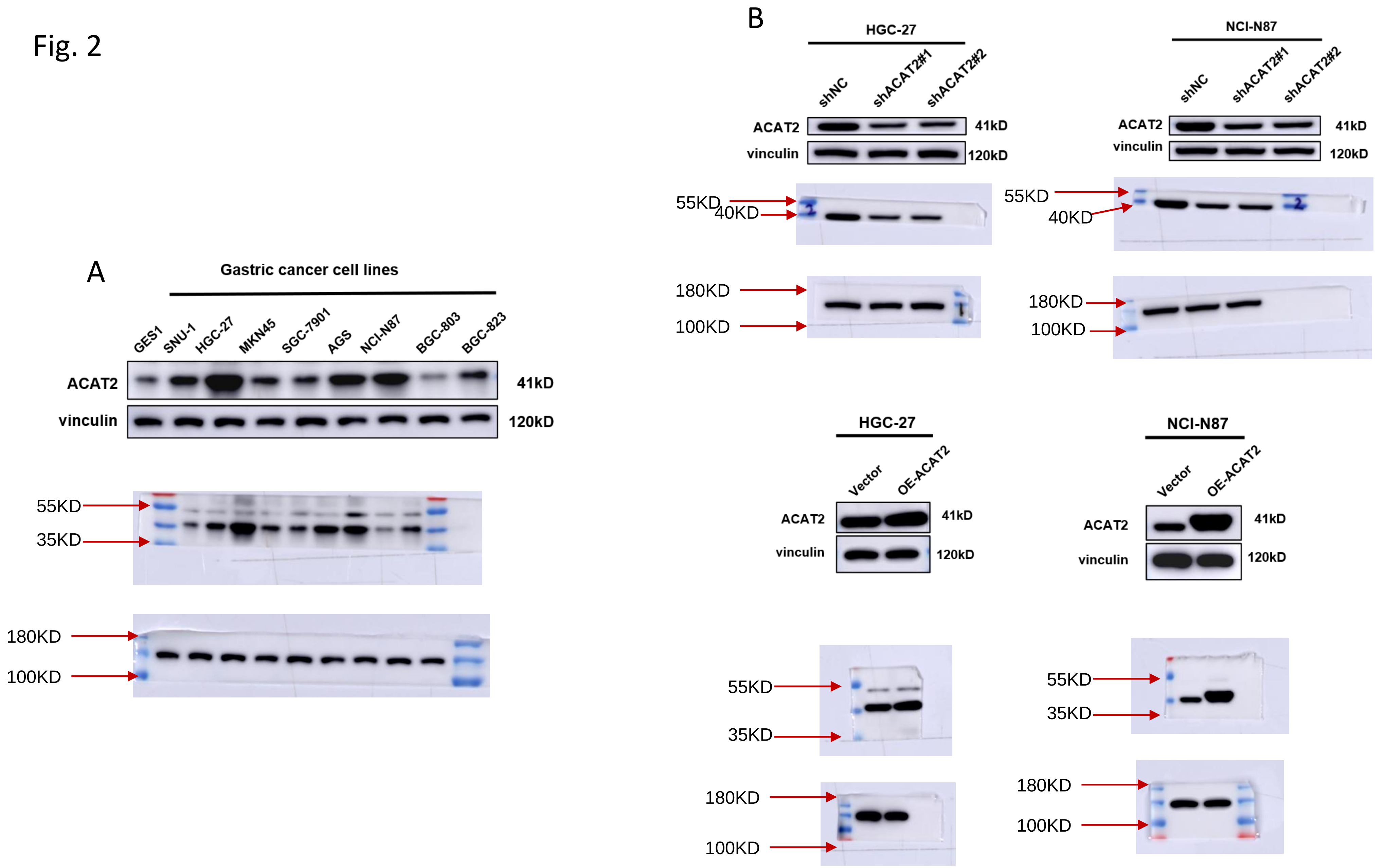

A
Gastric cancer cell lines
GES1
SNU-1
HGC-27
MKN45
SGC-7901
AGS
NCI-N87
BGC-803
BGC-823
ACAT2
41kD
vinculin
120kD
55KD
35KD
180KD
100KD
B
HGC-27
shNC
shACAT2#1
shACAT2#2
ACAT2
41kD
vinculin
120kD
NCI-N87
shNC
shACAT2#1
shACAT2#2
ACAT2
41kD
vinculin
120kD
55KD
40KD
55KD
40KD
180KD
100KD
180KD
100KD
HGC-27
Vector
OE-ACAT2
ACAT2
41kD
vinculin
120kD
NCI-N87
Vector
OE-ACAT2
ACAT2
41kD
vinculin
120kD
55KD
35KD
55KD
35KD
180KD
100KD
180KD
100KD

Fig. 2C

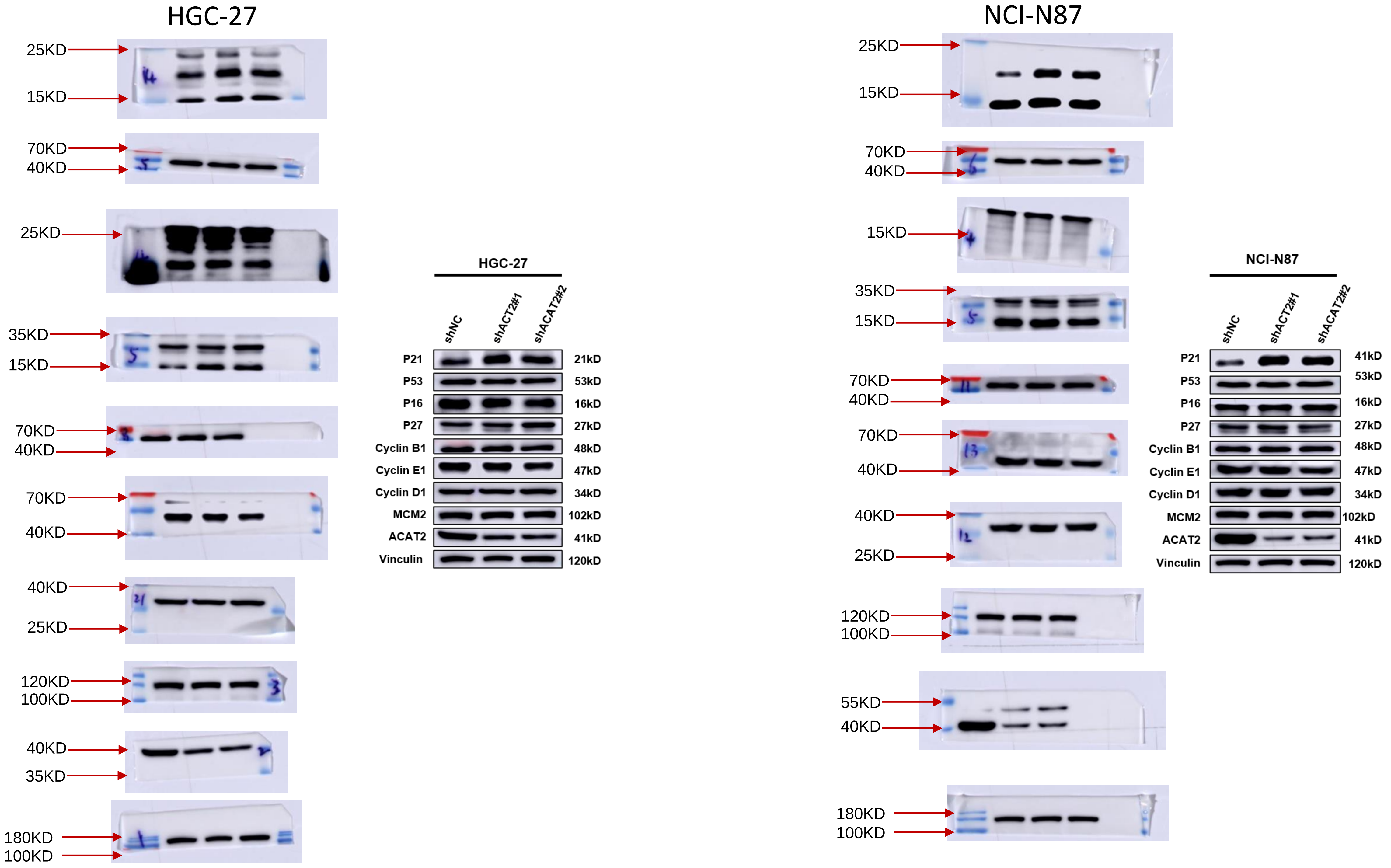

HGC-27
25KD
15KD
70KD
40KD
25KD
35KD
15KD
70KD
40KD
70KD
40KD
40KD
25KD
120KD
100KD
40KD
35KD
180KD
100KD
HGC-27
shNC
shACT2#1
shACAT2#2
P21
21kD
P53
53kD
P16
16kD
P27
27kD
Cyclin B1
48kD
Cyclin E1
47kD
Cyclin D1
34kD
MCM2
102kD
ACAT2
41kD
Vinculin
120kD
NCI-N87
25KD
15KD
70KD
40KD
15KD
35KD
15KD
70KD
40KD
70KD
40KD
40KD
25KD
120KD
100KD
55KD
40KD
180KD
100KD
NCI-N87
shNC
shACT2#1
shACAT2#2
P21
41kD
P53
53kD
P16
16kD
P27
27kD
Cyclin B1
48kD
Cyclin E1
47kD
Cyclin D1
34kD
MCM2
102kD
ACAT2
41kD
Vinculin
120kD

Fig. 3 EMT

AGS

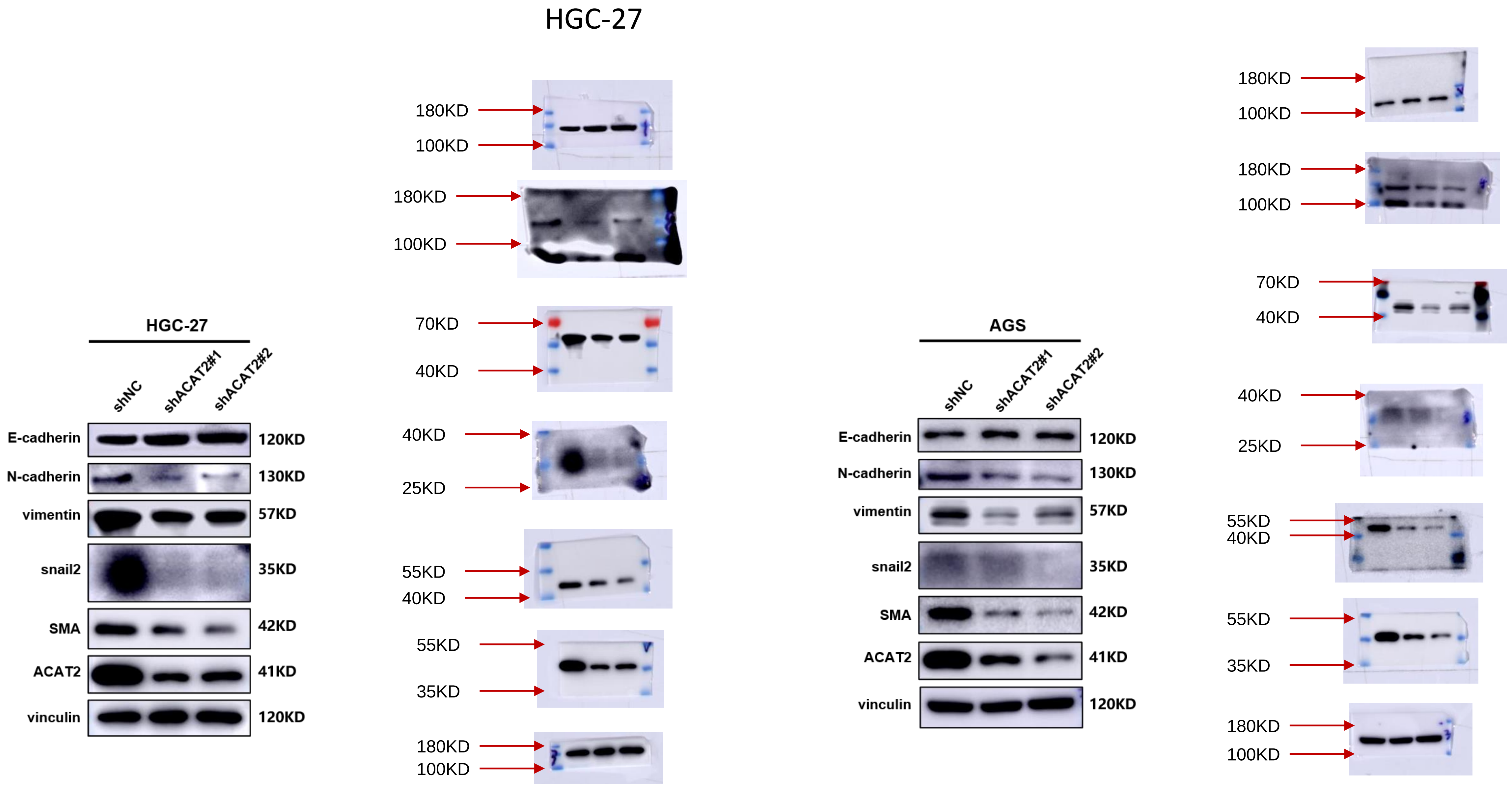
HGC-27
180KD
100KD
180KD
100KD
70KD
40KD
40KD
25KD
55KD
40KD
55KD
35KD
180KD
100KD
HGC-27
shNC
shACAT2#1
shACAT2#2
E-cadherin
120KD
N-cadherin
130KD
vimentin
57KD
snail2
35KD
SMA
42KD
ACAT2
41KD
vinculin
120KD
AGS
shNC
shACAT2#1
shACAT2#2
E-cadherin
120KD
N-cadherin
130KD
vimentin
57KD
snail2
35KD
SMA
42KD
ACAT2
41KD
vinculin
120KD
180KD
100KD
180KD
100KD
70KD
40KD
40KD
25KD
55KD
40KD
55KD
35KD
180KD
100KD

Fig. 4E

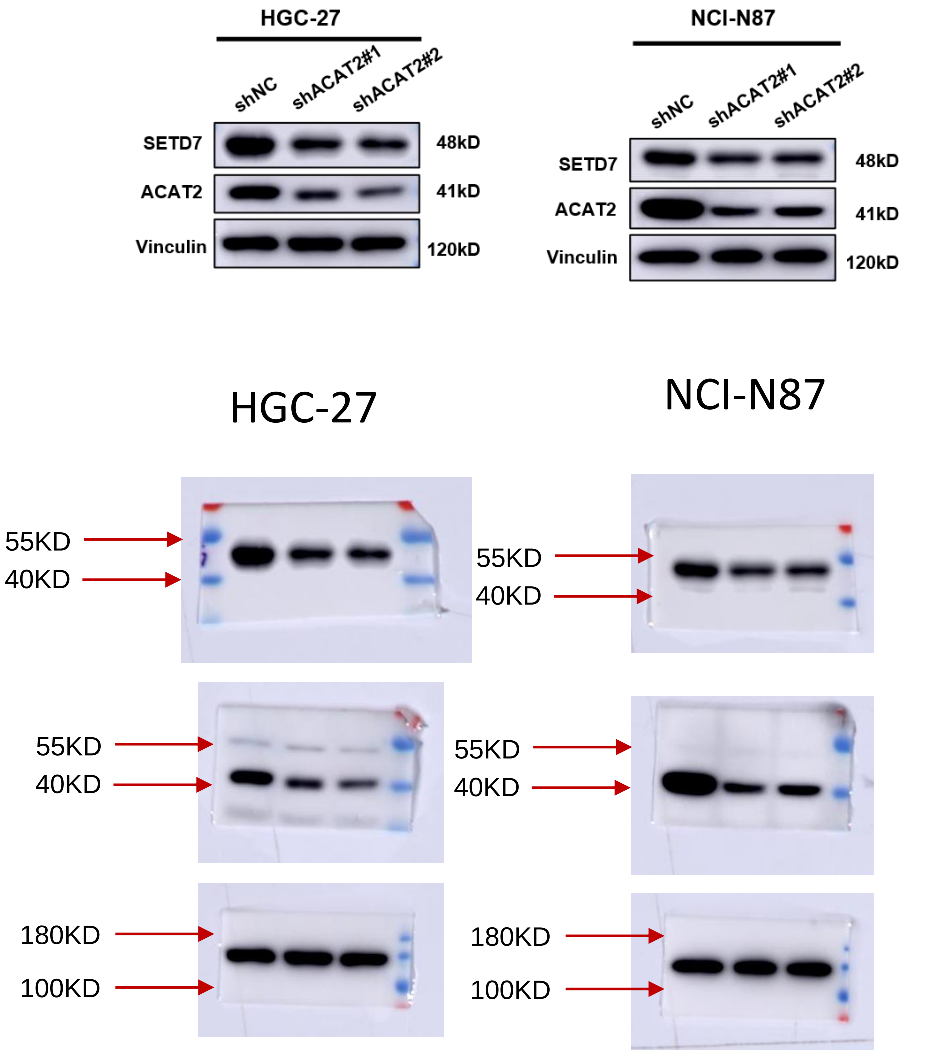

Fig. 5D

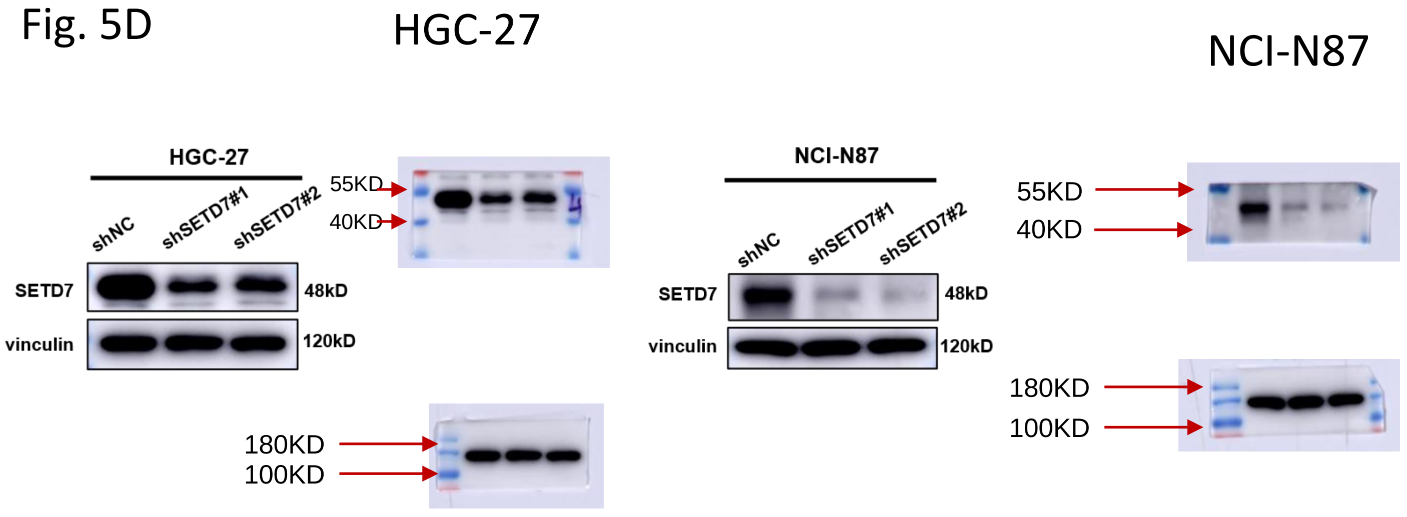

Fig. 6B

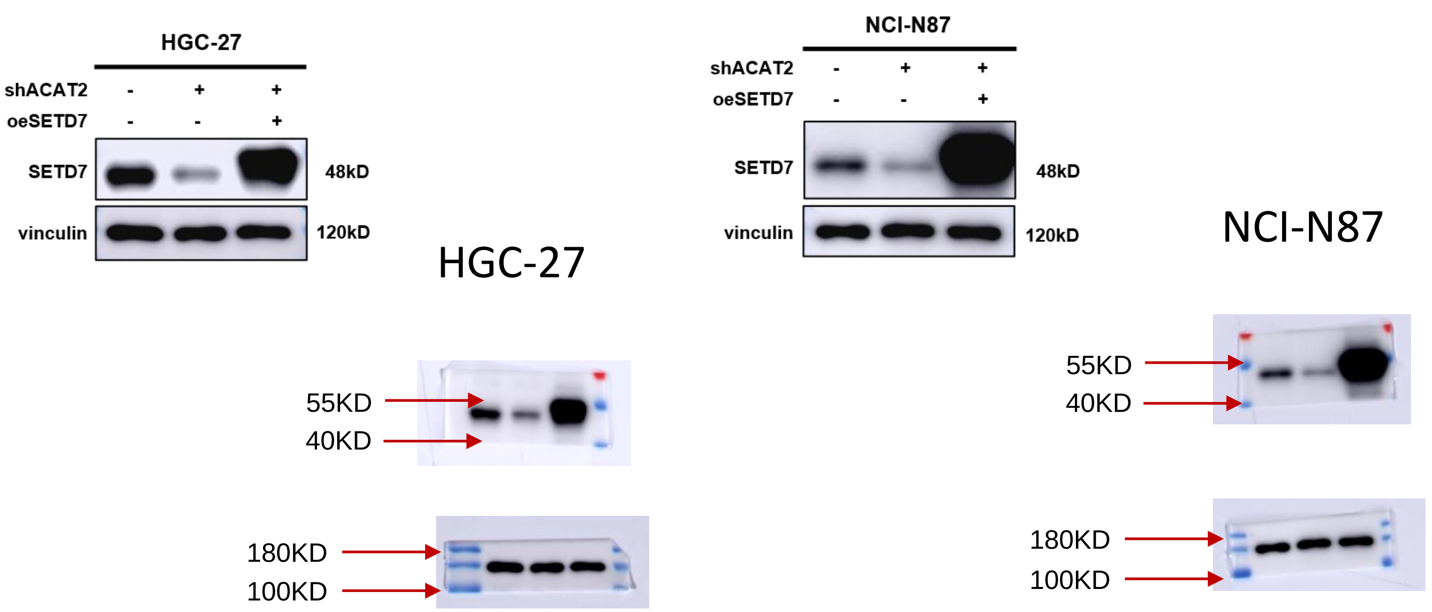

Fig. 7B

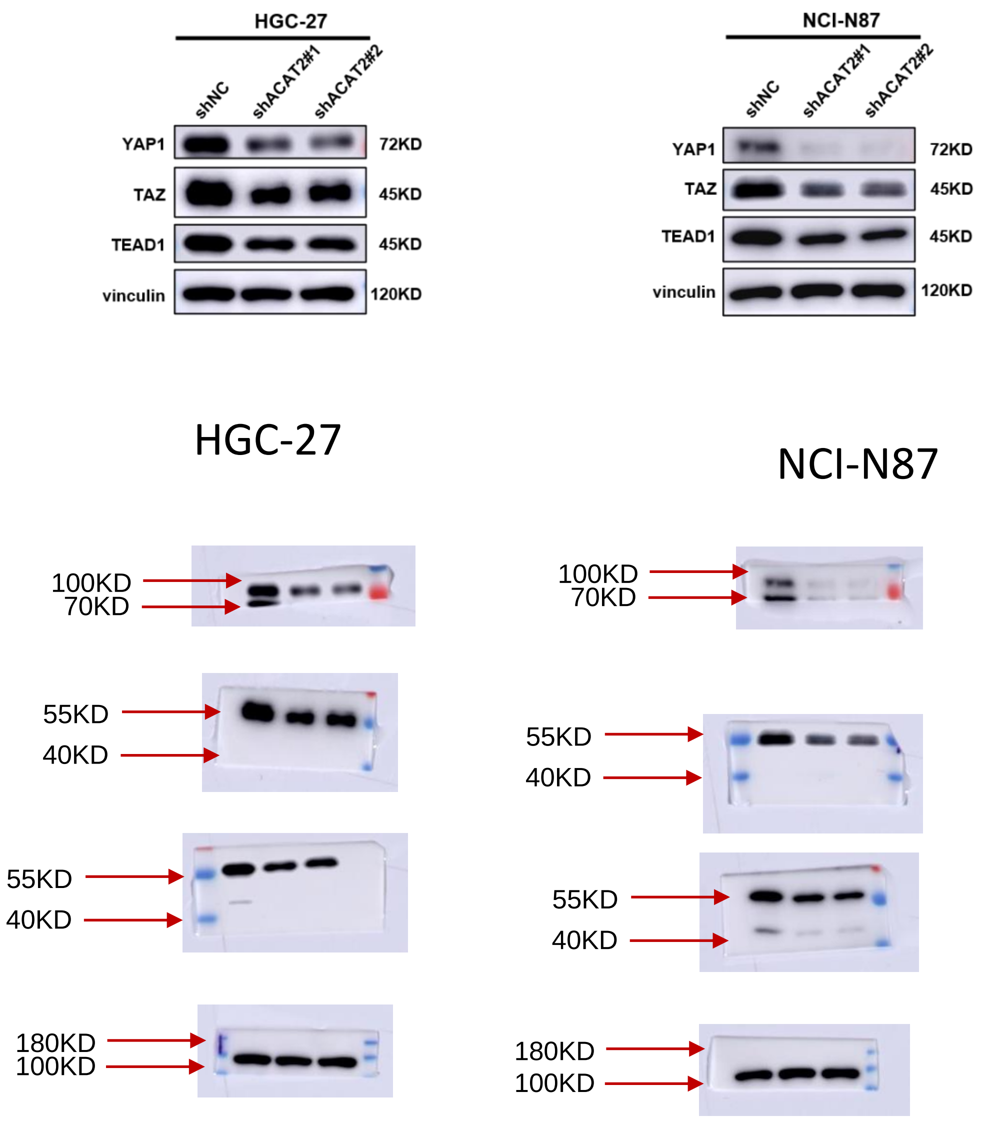

HGC-27
shNC
shACAT2#1
shACAT2#2
YAP1
TAZ
TEAD1
vinculin
72KD
45KD
45KD
120KD
NCI-N87
shNC
shACAT2#1
shACAT2#2
YAP1
TAZ
TEAD1
vinculin
72KD
45KD
45KD
120KD
HGC-27
100KD
70KD
55KD
40KD
55KD
40KD
180KD
100KD
NCI-N87
100KD
70KD
55KD
40KD
55KD
40KD
180KD
100KD

Fig. 7C

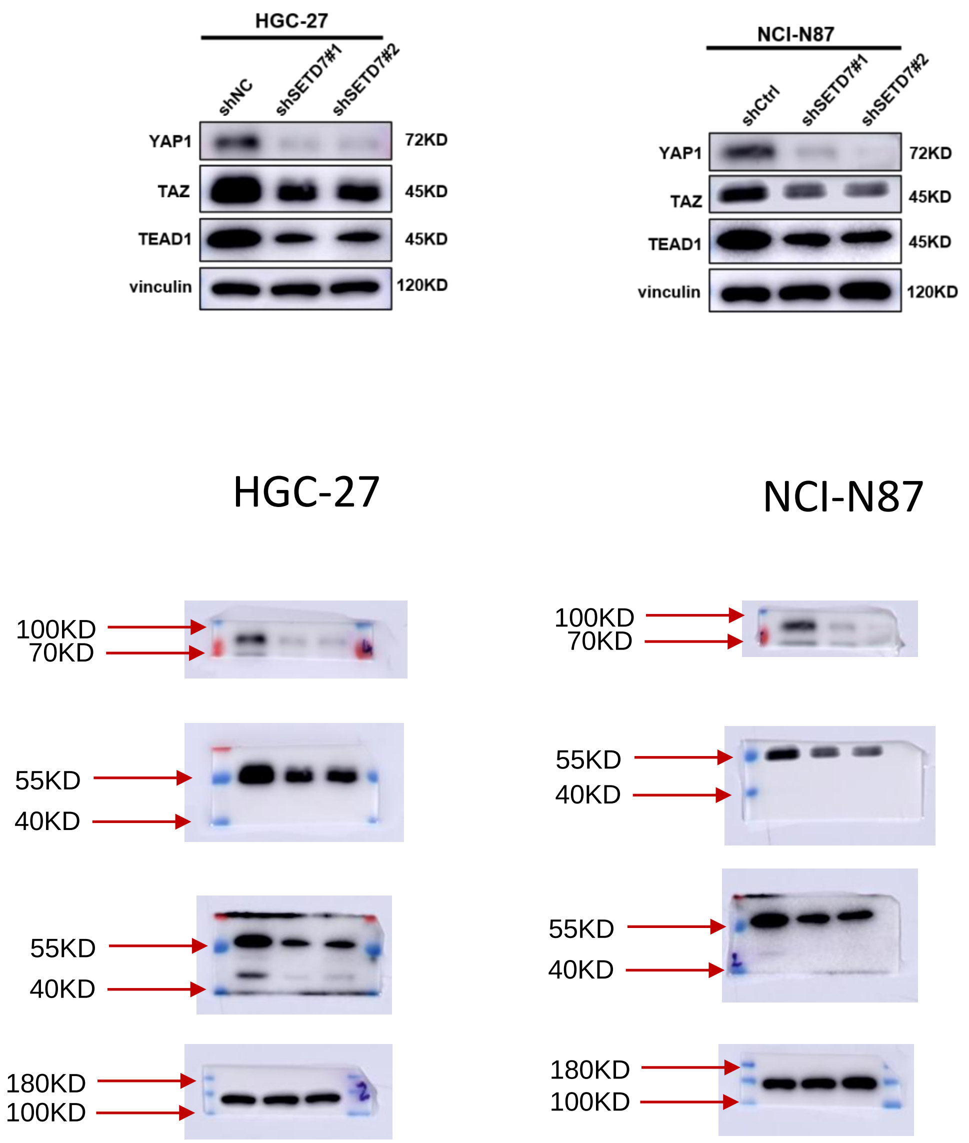

HGC-27
shNC
shSETD7#1
shSETD7#2
YAP1
TAZ
TEAD1
vinculin
72KD
45KD
45KD
120KD
NCI-N87
shCtrl
shSETD7#1
shSETD7#2
YAP1
TAZ
TEAD1
vinculin
72KD
45KD
45KD
120KD
HGC-27
100KD
70KD
55KD
40KD
55KD
40KD
180KD
100KD
NCI-N87
100KD
70KD
55KD
40KD
55KD
40KD
180KD
100KD

Fig. 7D

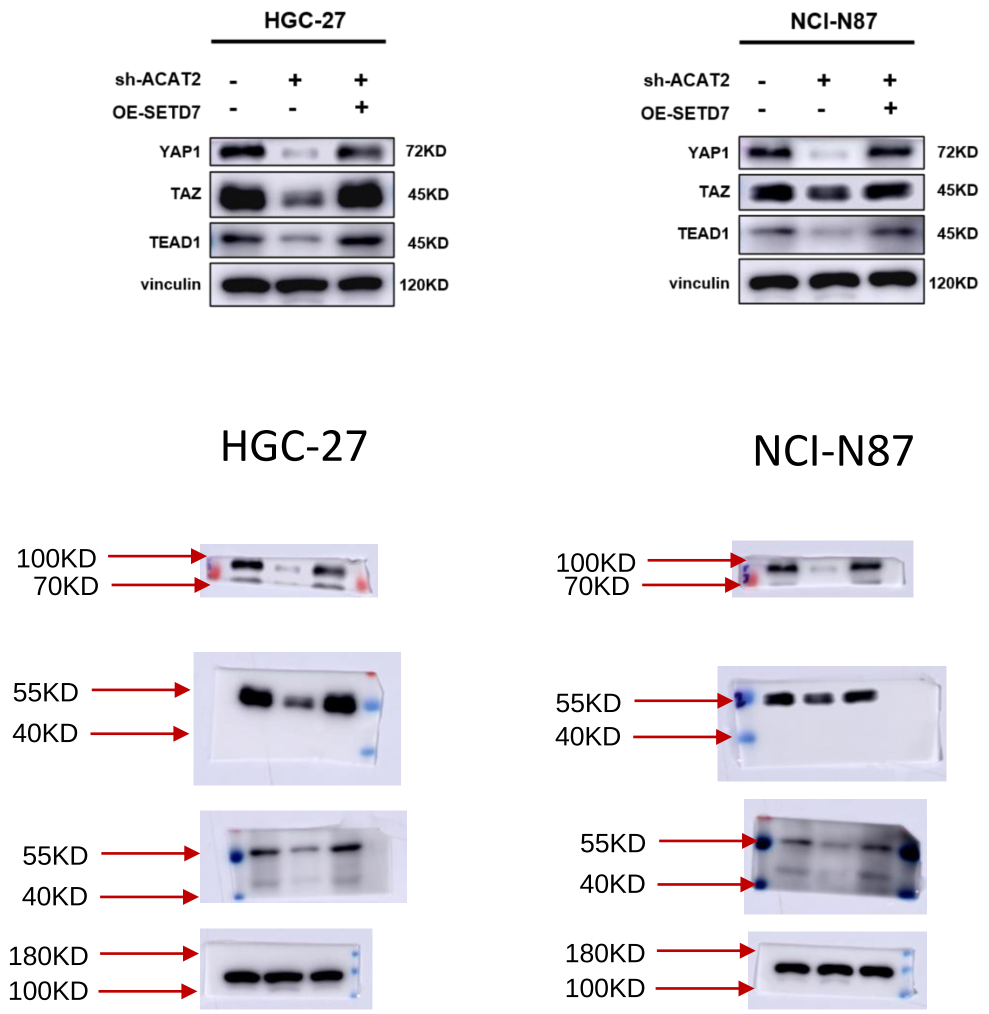
HGC-27
sh-ACAT2 - + +
OE-SETD7 - - +
YAP1
72KD
TAZ
45KD
TEAD1
45KD
vinculin
120KD
NCI-N87
sh-ACAT2 - + +
OE-SETD7 - - +
YAP1
72KD
TAZ
45KD
TEAD1
45KD
vinculin
120KD
HGC-27
NCI-N87
100KD
70KD
55KD
40KD
55KD
40KD
180KD
100KD
100KD
70KD
55KD
40KD
55KD
40KD
180KD
100KD

Fig. 7E

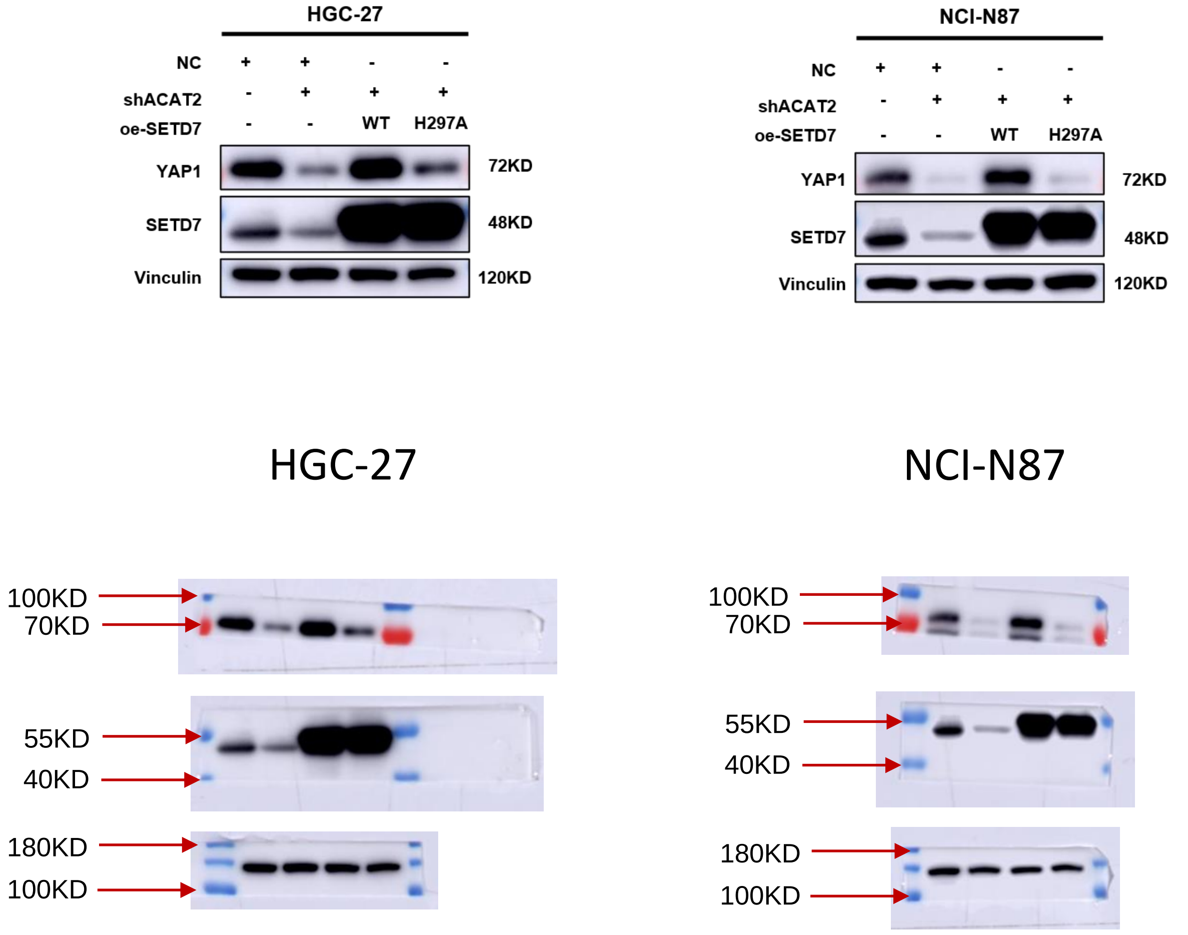
HGC-27
NC + + - -
shACAT2 - + + +
oe-SETD7 - - WT H297A
YAP1
72KD
SETD7
48KD
Vinculin
120KD
NCI-N87
NC + + - -
shACAT2 - + + +
oe-SETD7 - - WT H297A
YAP1
72KD
SETD7
48KD
Vinculin
120KD
HGC-27
NCI-N87
100KD
70KD
55KD
40KD
180KD
100KD
100KD
70KD
55KD
40KD
180KD
100KD

Fig. 7F

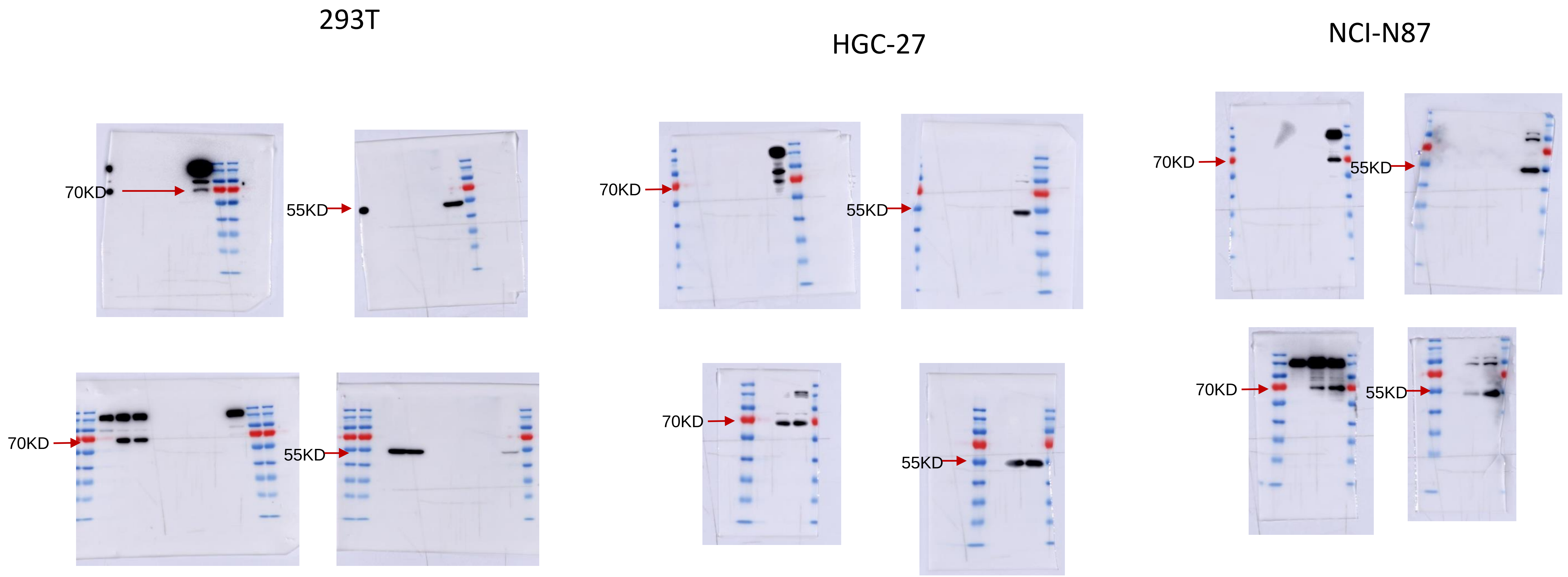
293T
HGC-27
NCI-N87
70KD
55KD
70KD
55KD
70KD
55KD
70KD
55KD
70KD
55KD
70KD
55KD

Fig. 7G

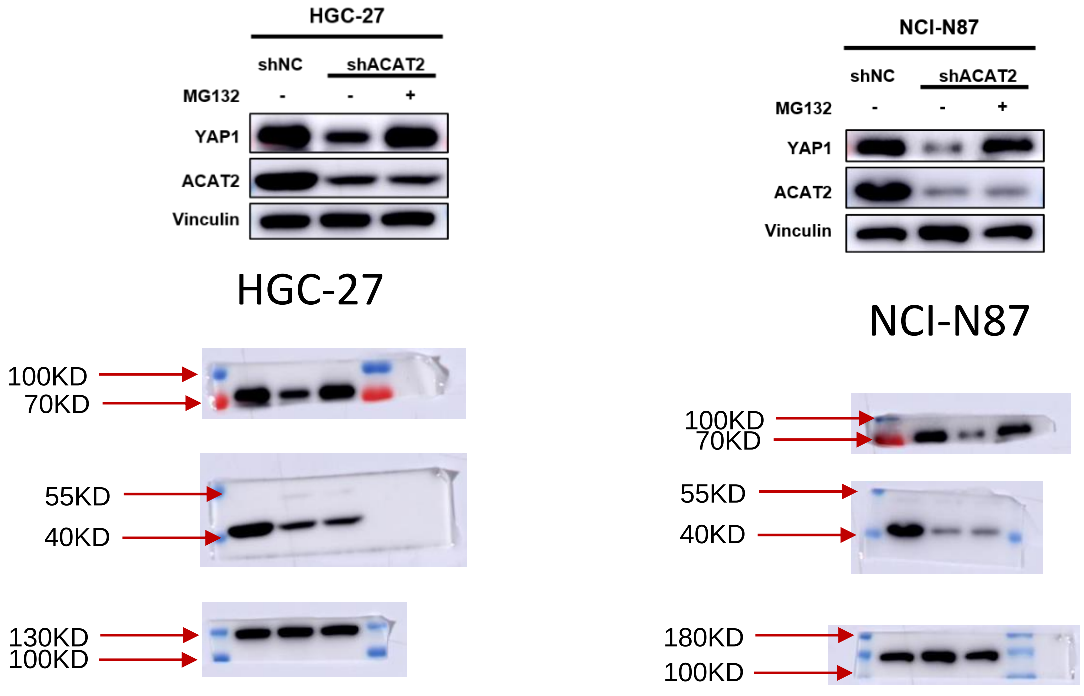

Fig. 7H

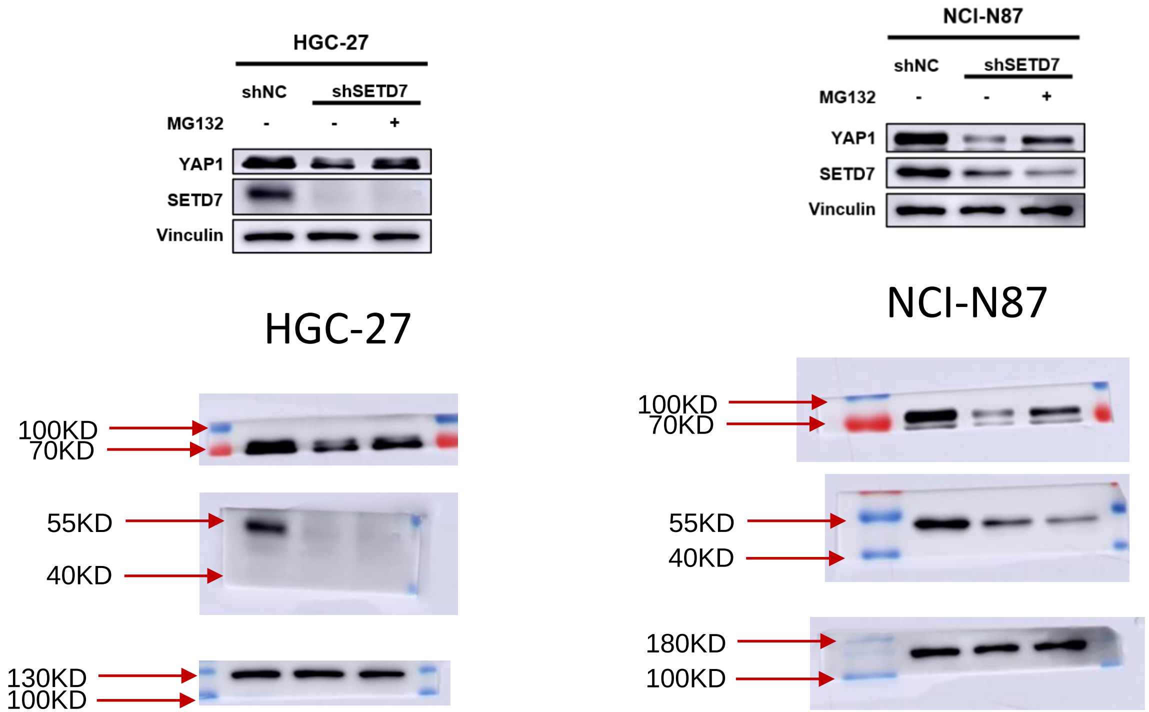

Fig. 7I

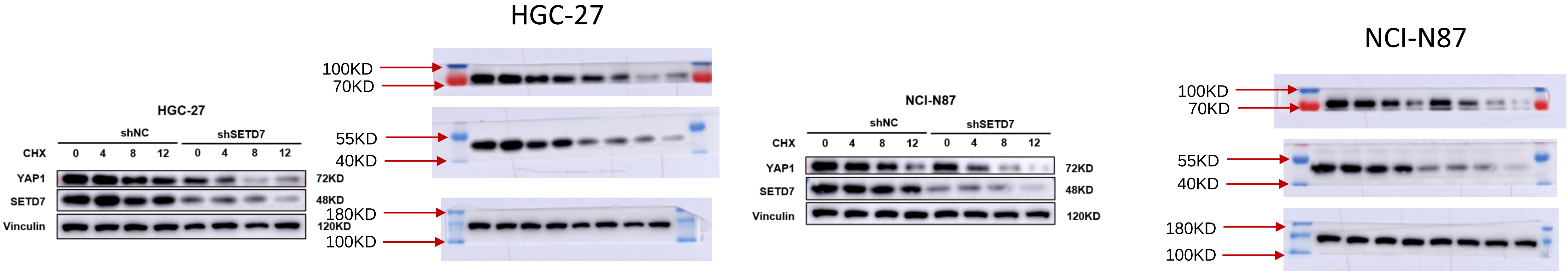

Fig. 7J

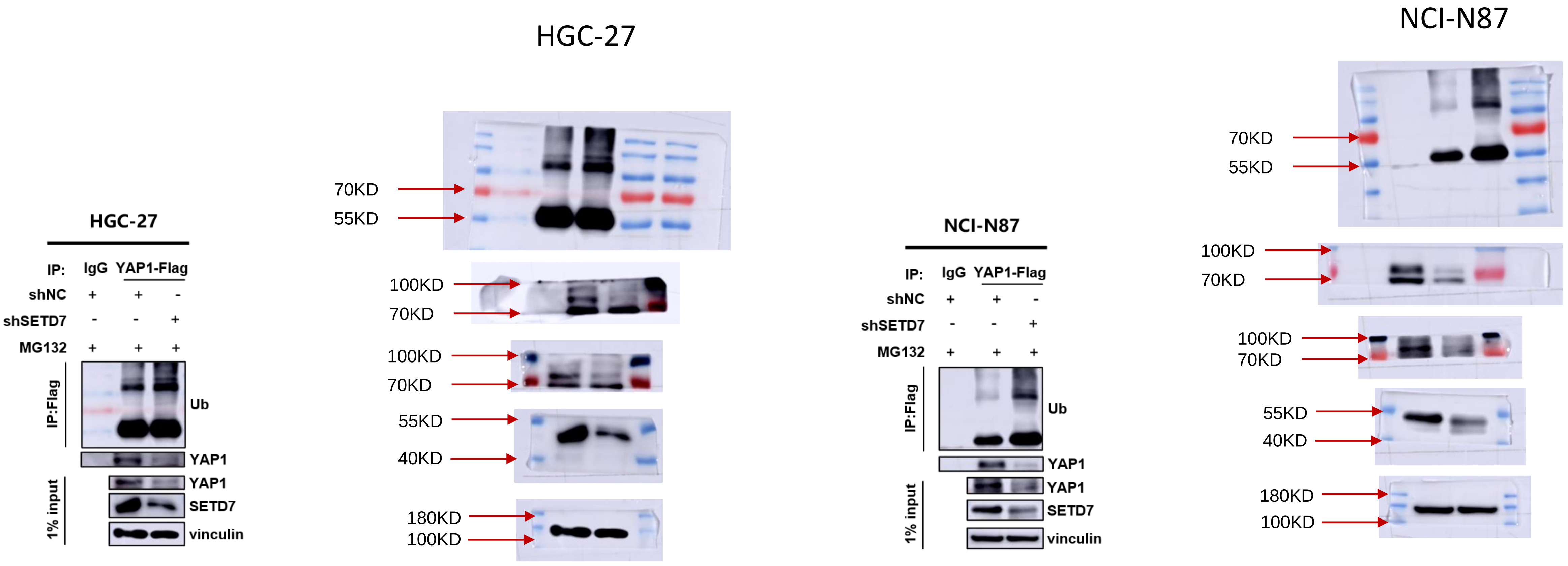
HGC-27
IP:
IgG
YAP1-Flag
shNC
shSETD7
MG132
+
-
IP:Flag
Ub
YAP1
1% input
YAP1
SETD7
vinculin
HGC-27
70KD
55KD
100KD
70KD
100KD
70KD
55KD
40KD
180KD
100KD
NCI-N87
IP:
IgG
YAP1-Flag
shNC
shSETD7
MG132
IP:Flag
Ub
YAP1
1% input
YAP1
SETD7
vinculin
NCI-N87
70KD
55KD
100KD
70KD
100KD
70KD
55KD
40KD
180KD
100KD

Fig S. 1B

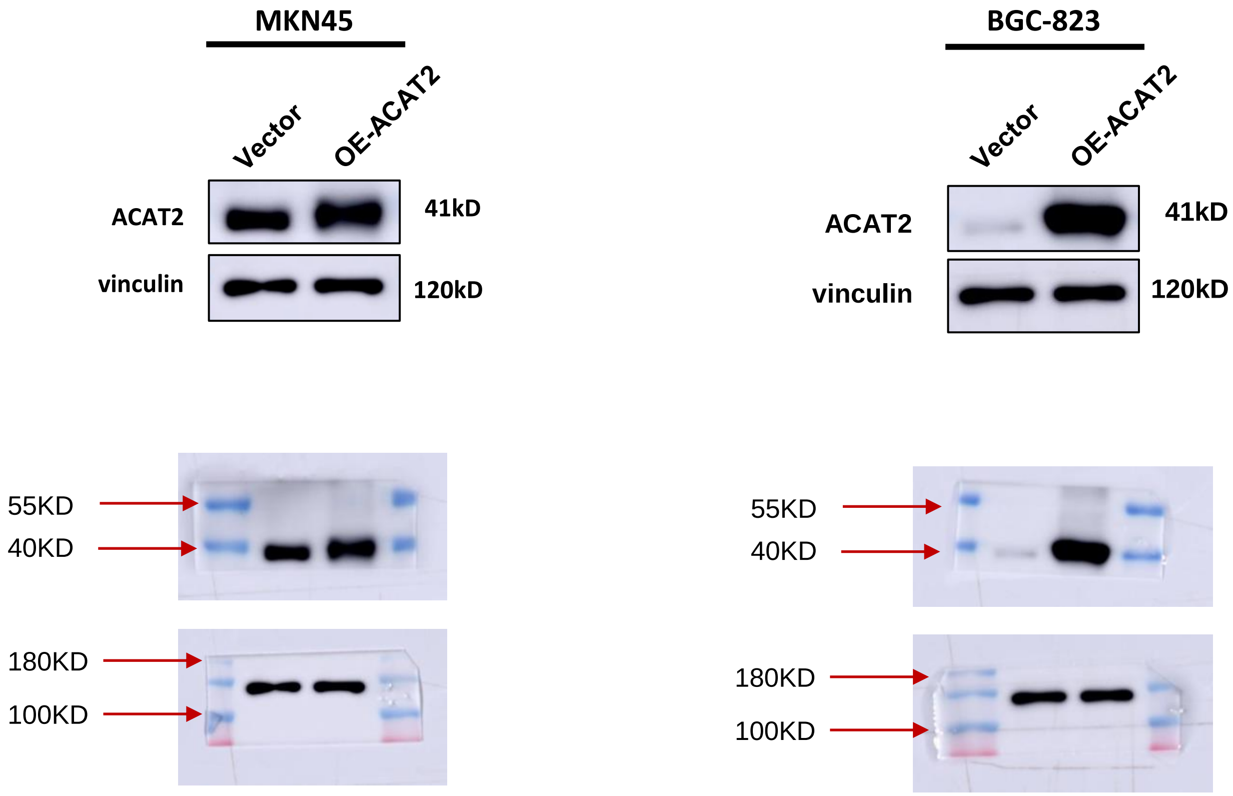
MKN45
Vector
OE-ACAT2
ACAT2
41kD
vinculin
120kD
BGC-823
Vector
OE-ACAT2
ACAT2
41kD
vinculin
120kD
55KD
40KD
180KD
100KD
55KD
40KD
180KD
100KD

Fig S. 3B

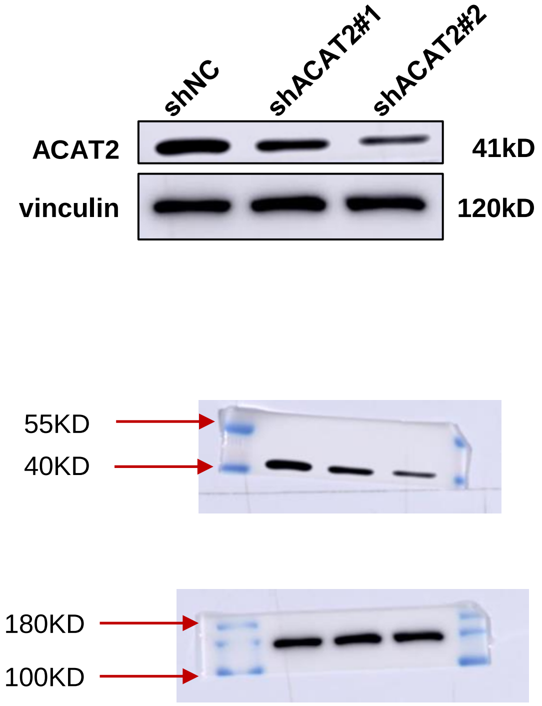
shNC
shACAT2#1
shACAT2#2
ACAT2
41kD
vinculin
120kD
55KD
40KD
180KD
100KD
